# From Elderflower to Bioactive Extracts: Phytochemical Characterization and Anti-Inflammatory Activity

**DOI:** 10.3390/molecules31030561

**Published:** 2026-02-05

**Authors:** Maryna Koval, Sławomir Dresler, Sandra Kowalik, Benedikt Schwarz, Anna Wawruszak, Anna Filipek, Małgorzata Komar, Thomas Jakschitz, Stavros Beteinakis, Günther Bonn, Wojciech Koch, Wirginia Kukula-Koch

**Affiliations:** 1 Department of Pharmacognosy with Medical Plants Garden, Medical University of Lublin, 1 Chodzki Str., 20-093 Lublin, Poland; marinchik.koval@gmail.com; 2 Department of Food and Nutrition, Medical University of Lublin, 4a Chodźki Str., 20-093 Lublin, Poland; sandrakowalik00@wp.pl (S.K.); kochw@interia.pl (W.K.); 3 Department of Analytical Chemistry, Medical University of Lublin, Chodźki 4a, 20-093 Lublin, Poland; slawomir.dresler@umlub.pl; 4 Department of Plant Physiology and Biophysics, Institute of Biological Sciences, Maria Curie-Skłodowska University, Akademicka 19, 20-033 Lublin, Poland; 5 Austrian Drug Screening Institute GmbH, Mitterweg 24, 6020 Innsbruck, Austria; benedikt.schwarz@adsi.ac.at (B.S.); anna.filipek@adsi.ac.at (A.F.); thomas.jakschitz@adsi.ac.at (T.J.);; 6 Department of Biochemistry and Molecular Biology, Medical University of Lublin, Chodzki Str. 1, 20-093 Lublin, Poland; anna.wawruszak@umlub.pl; 7 Department of Animal Anatomy and Histology, University of Life Sciences, Akademicka Str. 12, 20-033 Lublin, Poland; malgorzata.matysek@up.edu.pl; 8 Division of Pharmacognosy and Natural Products Chemistry, Department of Pharmacy, National and Kapodistrian University of Athens, Panepistimioupoli Zografou, 15771 Athens, Greece; sbeteinakis@pharm.uoa.gr

**Keywords:** elderberry, HPLC-MS fingerprinting, anti-inflammatory potential, extraction, chemometrics, HaCaT NF-κB Luc reporter cells, cytotoxic properties

## Abstract

This study provides a phytochemical characterization of *Sambucus nigra* L. (elderflower) and correlates its chemical profile with anti-inflammatory bioactivity, establishing an optimized extraction methodology. A comparative analysis of ultrasound-assisted extraction (UAE), accelerated solvent extraction (ASE), and shaking maceration was conducted using solvents of varying polarity (ethanol, ethanol–water mixture (1:1, *v*/*v*), and water). High-resolution fingerprinting via HPLC-ESI-QTOF-MS/MS confirmed a rich polyphenolic profile, dominated by flavonoids such as rutin, naringenin, and phenolic acids, notably chlorogenic acid. Quantitative analysis revealed that UAE with ethanol–water mixture (1:1, *v*/*v*) for 20 min yielded the highest recovery of rutin (4.87%) and chlorogenic acid (8.22%). The anti-inflammatory potential was evaluated in TNFα-stimulated HaCaT NF-κB Luc reporter keratinocytes. Anhydrous ethanolic extracts demonstrated superior efficacy, significantly inhibiting NF-κB pathway activation at non-cytotoxic concentrations. Chemometric analysis, specifically PLS-DA, identified naringenin as a principal contributor to this observed anti-inflammatory effect. These findings underscore the critical role of solvent selection in modulating the phytochemical composition and resultant bioefficacy of elderflower extracts. The potent, naringenin-driven inhibition of NF-κB in keratinocytes highlights the significant therapeutic potential of optimized *S. nigra* extracts for applications in dermatological and cosmetic formulations aimed at managing inflammatory skin disorders.

## 1. Introduction

The genus *Sambucus* L. (family Viburnaceae) comprises a group of flowering plants commonly known as elder or elderberry. It includes shrubs, small trees, and herbaceous species distributed mainly across temperate and subtropical regions of the Northern Hemisphere; in addition, some species are common in Asia, North America, New Zealand and Australia [1]. Among them, *Sambucus nigra* L. (European elder) is the most widely studied and utilized species. Traditionally, various parts of the plant—including flowers, fruits, bark, and leaves—have been employed in folk medicine for their diaphoretic, diuretic, laxative, and anti-inflammatory effects. Moreover, *S. nigra* is officially recognized and included in several pharmacopoeias worldwide [2].

Phytochemical studies have revealed that elderflowers are rich in flavonoids, primarily rutin and naringenin. In addition, they contain other flavonoids, including isoquercitrin, astragalin, hyperoside, nicotiflorin, and isorhamnetin glycosides, as well as kaempferol and quercetin aglycones in smaller amounts. Among phenolic acids, chlorogenic acid plays a particularly important role, accompanied by dicaffeoylquinic acid and 5-*p*-coumaroylquinic acid [2,3].

Rutin is a quercetin glycoside found in various plants and fruits. Particularly rich sources include members of the Polygonaceae family (common buckwheat, *Rheum* spp., *Rumex* spp.), as well as citrus fruits, elderflowers, green tea, and others. This flavonoid has a wide field of properties: antioxidant, anti-inflammatory, neuroprotective and anticancer [4,5]. Naringenin, better known as a citrus flavonoid, displays strong anti-inflammatory and antioxidant activities and is used as a supplement in the treatment of obesity, diabetes, and hypertension [6]. Another important phenolic compound abundant in elderberry flowers is chlorogenic acid. This widespread substance exhibits therapeutic effects similar to those of flavonoids in chronic metabolic diseases and age-related disorders [7]. As shown above, the presence of a wide variety of polyphenols in the extracts explains the traditional uses of elderberry flowers and represents a multitude of potential applications in phytotherapy and medicine. As the specialized metabolites synthesized by the plant come from different subgroups of organic compounds and are characterized by varying polarity, it is of the highest importance to be aware of the extraction conditions that should be applied to recover them from plant matrix.

The choice of extraction techniques, solvent type, duration and temperature plays a key role in preserving and maximizing the yield of phenolic compounds from *S. nigra* flowers; since they are primarily responsible for antioxidant activity and significantly contribute to the therapeutic potential, the choice of the most appropriate extraction method is crucial in obtaining extracts with optimal pharmacological properties. In spite of the rising interest in green extraction approaches and their role in modulating bioactive compounds, there is still a lack of detailed information concerning the influence of extraction methods and environmentally friendly solvents on the phytochemical composition and activity of *S. nigra* flowers. Therefore, the aim of this study was to evaluate the efficacy of various extraction techniques (ultrasound-assisted extraction—UAE, shaking maceration—SM, and accelerated solvent extraction—ASE) and solvents (ethanol, ethanol–water mixture (1:1, *v*/*v*), and water) on the chemical composition of the obtained extracts. This study also focused on the identification of the major natural products present in the elderflower extracts using an HPLC-ESI-QTOF-MS/MS approach and sought to establish the optimal extraction strategy by combining the appropriate extraction technique, duration, and solvent, leading to the most efficient recovery of phenolic compounds. In addition, the cytotoxic and anti-inflammatory potential of selected extracts was estimated using HaCaT NF-κB Luc reporter cells to evaluate biological potential. Furthermore, *S. nigra* flowers of Ukrainian origin were investigated to determine whether extraction methods previously optimized for Polish plant material would provide comparable yields of key phenolic antioxidants, such as rutin and chlorogenic acid, when applied to a different geographical source. This approach allows us to assess both the reproducibility of the extraction strategies and the potential impact of raw material origin on the phenolic profile. The results of this work will provide valuable information for the development and standardization of evidence-based phytopharmaceuticals, cosmetic products, and food formulations derived from *S. nigra* flowers [8].

Taken together, the present study extends current knowledge on *Sambucus nigra* flower extracts by combining systematic extraction optimization, high-resolution metabolite fingerprinting, advanced chemometric analysis, and functional validation in a mechanistically relevant NF-κB reporter keratinocyte model. This integrative approach reveals previously unreported qualitative–quantitative relationships among extraction strategy, metabolite composition, and anti-inflammatory bioactivity.

## 2. Results

### 2.1. Quantitative Analysis of the Obtained Extracts by HPLC-PDA Chromatography

First, the HPLC analysis of *S. nigra* flower extracts was performed to determine the content of the main phenolic constituents of elderberry flower, rutin and chlorogenic acid, under the influence of different extraction parameters. Their quantification in the obtained extracts was performed using HPLC-PDA instrumentation.

**Figure 1:**
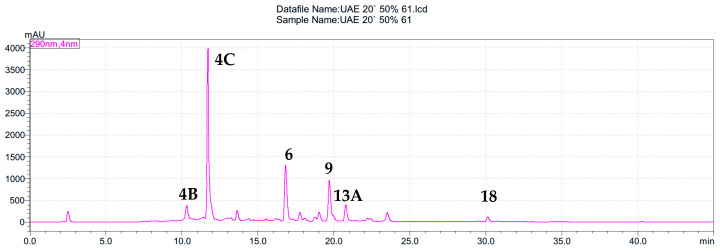
HPLC chromatogram of *S. nigra* flower extracts obtained by the ultrasound-assisted extraction with EtOH–H_2_O mixture (1:1, *v*/*v*) (extract 61) at 290 nm. The peaks are numbered according to the compounds listed in Table 2.

As shown in Figure 1, in Table 1, and in the extracts’ fingerprints (Supplementary File, Figures S2–S5), the number of rutin and chlorogenic acid compounds depends on the extraction method, solvent, and geographical origin of the samples. However, the intensity and frequency of peak appearance in the chromatograms were more similar for each type of solvent (ethanol to ethanol, water to water, etc.) than for the extraction method, which will be further discussed in the section focusing on the chemometric analysis of the raw data. The similarity of the chromatographic profiles obtained with the same extracting solvent can be explained by the fact that the solvent determines the chemical composition of the extract, whereas the extraction method mainly affects its quantitative parameters. Each solvent (water, ethanol, and ethanol–water mixture (1:1, *v*/*v*)) has a specific polarity and accordingly dissolves a specific range of compounds. Therefore, irrespective of the extraction method, the set of main compounds transferred into the solution remained similar. A sample HPLC-PDA fingerprint from the study is presented in Figure 1, whereas the table with the calculated rutin and chlorogenic acid contents (Table 1) follows.

According to Table 1, the highest rutin content was recovered by the UAE extraction for 20 min with the ethanol–water mixture (1:1, *v*/*v*), yielding a concentration of 4.87% (n = 3). The greatest amount of chlorogenic acid was also detected under the same conditions, with a mean value of 8.22% (n = 3). Thus, this extraction protocol proved to be the most effective for simultaneously extracting both target compounds. Among the shaking-assisted extractions, the highest rutin (3.20%) and chlorogenic acid (4.71%) contents were obtained in extract 17, prepared using an ethanol–water mixture (EtOH-H_2_O), with an extraction time of 10 min. In the case of ASE extraction, the highest rutin content (3.25%) was detected in extract 42, prepared with ethanol for 15 min at 100 °C. The second-highest value of chlorogenic acid (5.01%) was observed in extract 25 under ASE conditions using an ethanol–water (EtOH–H_2_O) solvent system with an extraction time of 5 min at 100 °C. High contents of both rutin (3.46%) and chlorogenic acid (4.77%) were also observed in extract 1, obtained using an ethanol–water (EtOH–H_2_O) solvent system with an extraction time of 5 min The chlorogenic acid yield was higher than that of rutin, indicating that the extract samples were richer in this compound under the applied extraction conditions. In terms of solvents, hydroethanolic mixtures (EtOH–H_2_O mixture (1:1, *v*/*v*) were superior to pure ethanol or water, providing the highest recovery of these two quantified phenolic compounds. In ASE, temperature significantly affected extraction efficiency, with 100 °C generally providing the highest yields of rutin and chlorogenic acid, particularly at shorter extraction times. However, prolonged extraction at elevated temperature occasionally resulted in reduced compound levels, indicating possible thermal degradation or co-extraction of interfering components. Moreover, rutin appeared to be more sensitive to elevated temperatures, whereas chlorogenic acid exhibited higher thermal stability under the applied conditions. Overall, the ethanol–water (EtOH–H_2_O) solvent system generally resulted in higher mean contents of both compounds compared to the single solvents (water or ethanol), particularly in ultrasound-assisted extraction and shaking-assisted extraction. For ASE extraction, the use of an ethanol–water (EtOH–H_2_O) mixture led to higher rutin yields, while the highest chlorogenic acid contents were achieved using water. Time effects were method-dependent: shaking favored ~10 min, whereas UAE peaked around 20 min, with no consistent benefit from longer (30 min) processing.

The quantitative analysis revealed that the rutin and chlorogenic acid contents in the Ukrainian elderflower extracts differed from the levels previously determined in Polish material, even under identical extraction conditions (Table 1). In Ukrainian material, rutin ranged from 2.05% (ASE) to 2.93% (UAE), while in Polish samples, it reached up to 4.87%. Similarly, chlorogenic acid in Ukrainian elderflowers ranged from 3.74% (ASE) to 5.00% (UAE), compared to 8.22% in the Polish samples.

### 2.2. The Compositional Analysis of Sambucus nigra Flower Extract by HPLC-ESI-QTOF-MS/MS

**Figure 2:**
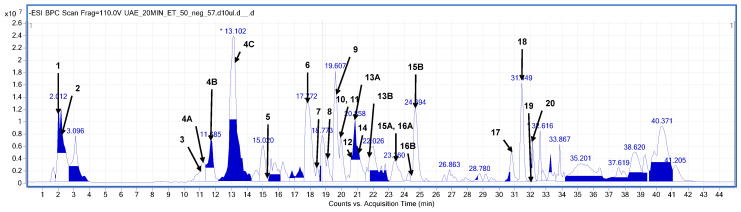
Base peak chromatogram (BPC) obtained in the negative ionization mode (HPLC-ESI-QTOF-MS/MS) from the elderberry flower extracted in ultrasounds for 20 min using EtOH–H_2_O mixture (1:1, *v*/*v*). The peaks are numbered according to the compounds listed in Table 2.

Compound assignment was based on high-resolution mass measurements, MS/MS fragmentation patterns (see Supplementary File Table S1), and previously published compositional data for this particular plant species. The analyzed plant material was found to be rich in a variety of components that were clearly visible, mainly in the negative ion mode, and well separated on the used chromatographic column. A sample mass chromatogram of one of the richest analyzed extracts is presented in Figure 2, below.

The extract’s fingerprinting revealed that the *S. nigra* flowers were particularly rich in polyphenolic constituents. The dominant compounds tentatively identified in the elderberry belonged mainly to different classes of flavonoids and phenolic acids (see Table 2).

Among flavonoids, rutin, quercetin 3-glucoside, kaempferol-3-*O*-rutinoside, and isorhamnetin-3-*O*-rutinoside were the major components, whereas naringenin, isorhamnetin pentoside, kaempferol derivative, isoquercitrin, isorhamnetin glucoside, and kaempferol glucoside were assigned as the components with lower concentration.

In addition, several phenolic acids, mainly those including the caffeic acid derivatives, like caffeoylquinic and dicaffeoylquinic acids, were detected in considerable amounts. Among this group, protocatechuic acid, coumaroylquinic acid, and coumaroyl-caffeoylquinic acid were assigned. Next to them, a vivid peak of quinic acid was observed. These compounds are well known for their pronounced antioxidant properties, which may significantly contribute to the extract’s overall antioxidant potential and may influence the total biological activity of the elderberry flower extracts. The analysis of the Ukrainian *S. nigra* flower extract revealed a chromatographic profile very similar to that of the Polish material (see Supplementary File Figure S1). The same main compounds were identified. This indicates that the qualitative composition of polyphenolic constituents was comparable across the two origins, suggesting a similar phytochemical pattern regardless of the tested raw material’s geographical origin. The chromatographic fingerprints of the registered extracts are presented in the Supplementary File (Figures S2–S5).

The phytochemical profile of the flower extracts tested in this study shows a correlation with previously published data; however, a larger number of molecules were identified in the substance assignment process described herein. The presence of key organic acids, numerous hydroxycinnamic acid derivatives (including multiple isomers of chlorogenic, coumaroylquinic, and dicaffeoylquinic acids), and a rich profile of flavonol glycosides (derivatives of quercetin, kaempferol, and isorhamnetin) was confirmed by the researchers, shedding light on the significant role of elderberry in delivering polyphenols. Phenolic acids, particularly esters derived from hydroxycinnamic acids, represent a quantitatively significant fraction of the *S. nigra* metabolome, with the highest concentrations observed in floral extracts.

Interestingly, the accomplished compounds assignment indicated the presence of two molecules—spermidine and pinellic acid—that are not often reported in this plant, but the registered fragmentation patterns and data on these metabolites in the scientific literature align with our data [17,19].

### 2.3. Impact of Solvent on the Composition of Extracts

The impact of extraction conditions on the recovery of chlorogenic acid and rutin, as described above, encouraged us to further investigate the effects of extraction conditions on other natural products present in the studied extracts. A series of injections on the UHPLC-MS chromatograph provided a rich data matrix for analyzing the impact of solvent on the extracts’ composition. As proven above, it was the solvent’s selection that showed the highest impact on the final shape of the registered fingerprint and the content of the analyzed components. Here, below, we present relative quantitative values obtained from the comparison of the chromatographic peak areas of the selected phenolic compounds across all obtained extracts.

**Figure 3:**
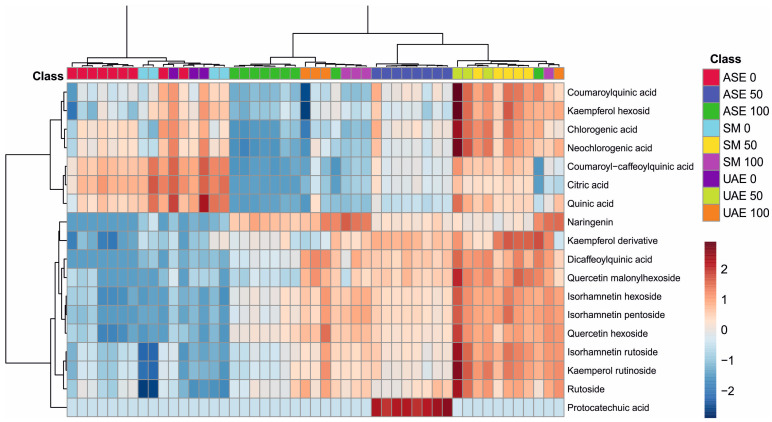
Heatmap showing the clustering of *S. nigra* flower extract samples using Ward’s method with Euclidean distance, illustrating differences in phenolic compound profiles depending on the extraction solvent and technique. The color scale ranges from dark blue (lowest relative abundance) to dark red (highest relative abundance) (the numbers 0, 50, and 100 correspond to water, EtOH-H_2_O and EtOH, whereas the abbreviations ASE, SM, and UAE stand for accelerated solvent extraction, shaking maceration, and ultrasound-assisted extraction). Color intensity reflects normalized peak area values, representing relative differences in compound abundance between extraction methods rather than absolute concentrations.

The analysis of metabolite profiles presented in the dendrogram and heatmap of Figure 3 clearly demonstrates that both the extraction technique and the polarity of the solvent used strongly modulated the qualitative and quantitative composition of the obtained extracts. The distinct clustering of samples along the horizontal axis reflects high consistency in metabolite profiles across the same combination of extraction method and ethanol concentration, confirming the decisive influence of extraction parameters on the efficiency of phenolic compound recovery.

The color patterns show that extracts obtained using high ethanol concentrations (100) were characterized by elevated levels of several more lipophilic metabolites, including isorhamnetin derivatives (hexoside, pentoside, rutinoside), kaempferol derivatives (including rutinoside), and selected dicaffeoylquinic acid derivatives. Conversely, water-based extracts and those obtained with lower ethanol concentrations (1:1 *v*/*v*) exhibited relatively high levels of more polar compounds, such as citric, quinic, chlorogenic, and neochlorogenic acids, consistent with their solubility profiles.

The extraction techniques themselves also differentiated the sample composition: notably, a characteristic clustering of ASE samples suggests a higher efficiency in recovering specific hydroxycinnamates, while UAE samples show increased extraction of certain flavonoids, likely due to cavitation-induced tissue disruption. SM, in contrast, presents a more balanced but less intense metabolite pattern, reflecting the milder nature of this extraction approach.

Overall, the figure confirms the strong, synergistic influence of ethanol concentration and extraction technique on the final composition of plant metabolites. The observed trends indicate that careful optimization of extraction conditions is essential for the targeted isolation of specific classes of compounds, and the clustering patterns provide a clear illustration of the selectivity inherent to each method.

**Figure 4:**
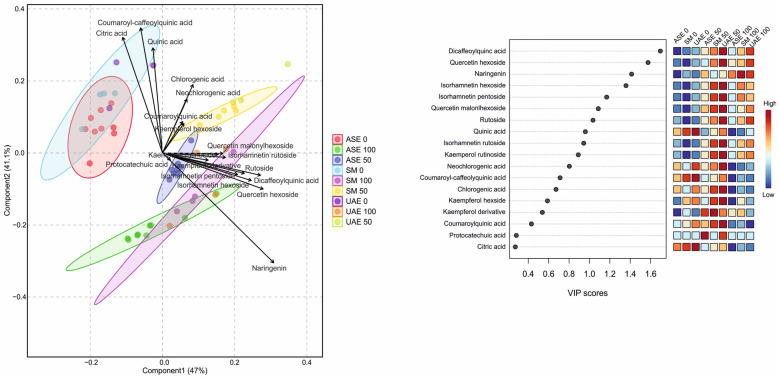
PLS-DA score plot (**left**) showing separation of *S. nigra* flower extracts according to extraction technique and ethanol concentration, together with the corresponding VIP scores and heatmap (**right**) identifying the metabolites most responsible for group discrimination. Higher VIP values indicate greater contribution of individual compounds to the model. The heatmap illustrates relative metabolite abundance across extraction conditions, with a color gradient from blue (low) to red (high).

The PLS-DA results presented in Figure 4 clearly demonstrate a strong separation of extracts obtained via accelerated solvent extraction (ASE) from those produced by shaking maceration (SM) and ultrasound-assisted extraction (UAE), indicating that ASE generates a distinct metabolite profile compared with the other techniques (see also Figure S6 in the Supplementary File). The first two components of the model explained 47% (Component 1) and 41.1% (Component 2) of the total variance. The robustness of the PLS-DA model was supported by cross-validation, yielding accuracy values of 0.366 (Component 1), 0.507 (Component 2), R-square values of 0.429 and 0.549, and Q-square predictability values of 0.338 and 0.447, confirming adequate model performance and predictive capacity. The loadings plot identifies citric acid, quinic acid, and coumaroyl–caffeoylquinic acid as the only metabolites that are strongly and positively associated with Component 2 and negatively with Component 1, which corresponds to the cluster of water-based extracts. In contrast, all remaining metabolites—predominantly flavonoid glycosides, as well as dicaffeoylquinic acids—are positively correlated with Component 1, which is mainly associated with ethanol-based extracts. This pattern aligns closely with the VIP score ranking, which highlights dicaffeoylquinic acid, quercetin hexoside, naringenin, and isorhamnetin glycosides as the most influential variables driving class separation. The heatmap of VIP-associated abundances further shows that ASE extracts—particularly those obtained at higher ethanol concentrations—are enriched in hydroxycinnamic acids (e.g., chlorogenic, neochlorogenic, coumaroylquinic acids), while SM and UAE extracts accumulate greater amounts of flavonoid glycosides, such as quercetin and isorhamnetin derivatives. Consistent with the previously discussed observations, solvent polarity exerts a pivotal influence on extraction selectivity, reinforcing that both the extraction technique and ethanol concentration jointly determine the dominant metabolite classes recovered in each extract.

The results of the analysis provided important insights into the behaviour of different metabolites under the extraction conditions. They were of particular importance for the studies on the anti-inflammatory potential of elderberry extracts that are presented in the section below.

### 2.4. Anti-Inflammatory Activity of the Selected Sambucus nigra Flower Extracts in Transfected HaCaT NF-κB Luc Reporter Cell Line

**Figure 5:**
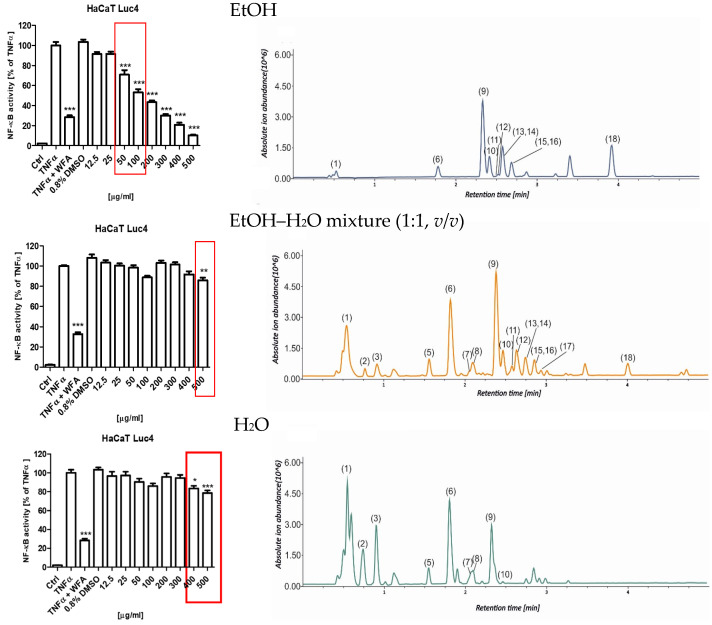
Effects of *S. nigra* flower extracts (EtOH (8), EtOH–H_2_O mixture (1:1, *v*/*v*) (9), and H_2_O (7) (12.5–500 µg/mL) on the activation of NF-κB in TNFα-treated HaCaT NF-κB Luc reporter cells. The data are presented as the mean ± standard deviation (±SD) of the mean. * *p* <  0.05, ** *p* <  0.01, *** *p* <  0.001 by one-way ANOVA, Tukey’s post hoc testing. Red frames—non-toxic, anti-inflammatory concentrations of *Sambucus nigra* flower extracts. The chromatograms represent the total ion chromatograms recorded in the UHPLC-MS analysis (1: quinic acid, 2: citric acid, 3: coumaroyl-caffeoylquinic acid isomer, 4: protocatechuic acid, 5: neochlorogenic acid, 6: chlorogenic acid, 7: kaempferol hexoside, 8: coumaroylquinic acid, 9: rutin, 10: quercetin hexoside, 11: quercetin malonylhexoside, 12: kaempferol rutinoside, 13: isorhamnetin rutoside, 14: isorhamnetin pentoside, 15: dicaffeoylquinic acid, 16: isorhamnetin hexoside, 17: kaempferol derivative, 18: naringenin).

To evaluate the anti-inflammatory potential of the *S. nigra* flower extracts, their capacity to inhibit TNFα-induced activation of NF-κB was assessed using a stably transfected HaCaT NF-κB Luc reporter cell line. This in vitro model is utilized for functional investigations of keratinocyte responses relevant to chronic inflammatory skin disorders and for screening anti-inflammatory compounds. This assay was performed in cells that were stimulated with 0.75 ng/mL TNFα or TNFα + WFA in the presence of either 0.8% dimethyl sulfoxide (DMSO; vehicle control) or 12.5–500 µg/mL *S. nigra* flower extracts. Extracts numbered (7, 8, and 9) assessed in this assay all came from UAE extraction for 20 min but were prepared using different solvents: EtOH (8), EtOH–H_2_O mixture (1:1, *v*/*v*) (9), and H_2_O (7). NF-κB-driven luciferase activity was subsequently quantified in cell lysates and compared across the tested extracts and treatment conditions (Figure 5). *S. nigra* EtOH extract decreased the NF-κB activity in a dose-dependent manner across the entire concentration range tested (Figure 5, top). However, only the concentrations of 50 and 100 µg/mL exhibited anti-inflammatory activity (70.88% and 53.11%, respectively) while simultaneously demonstrating no cytotoxic effects on keratinocytes. Reduced anti-inflammatory activity, albeit less pronounced than for the EtOH extract, was observed for extract 9 at concentrations of 400 (83.50%) and 500 µg/mL (78.63%) (Figure 5, middle) and for extract 7 at 500 µg/mL (85.89%) (Figure 5, bottom).

Figure 5 shows a dependence between the extraction solvent used and the extract fingerprint, which affects bioactivity. EtOH, as a non-aqueous extracting solvent, does not recover from the plant matrix polar compounds, which are visible at the beginning of the presented chromatograms. The most active extract contains the highest quantities of compounds eluted after an Rt of 3 min, which were assigned as Compounds **9**–**16** and **18**.

**Figure 6:**
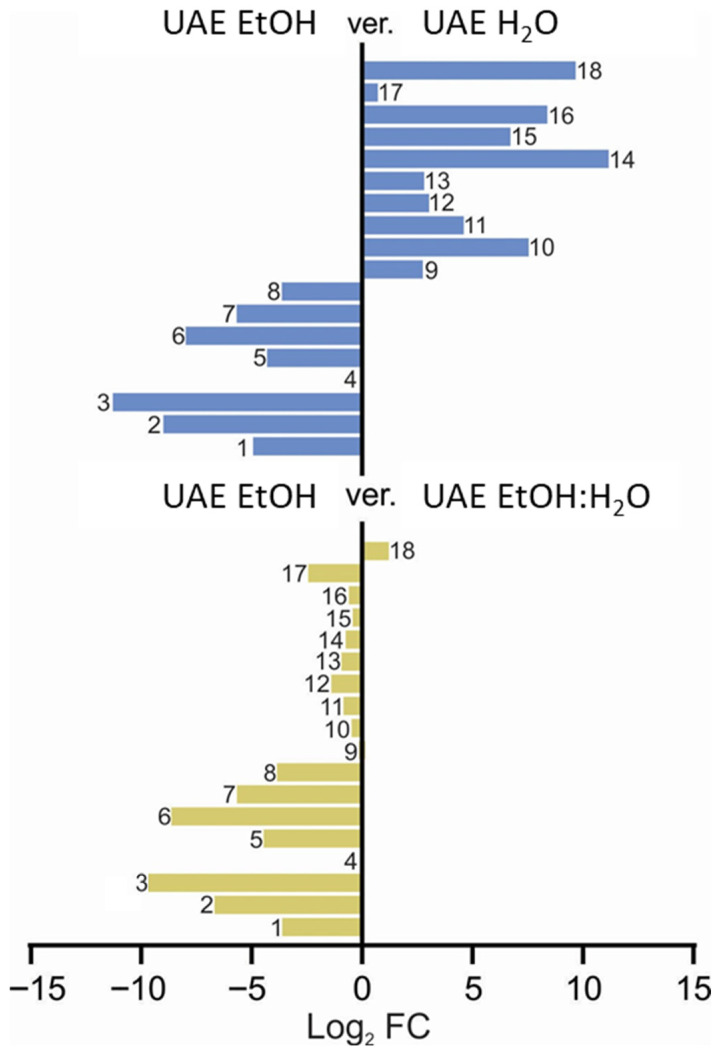
Qualitative comparison of fingerprints of the extracts studied for their anti-inflammatory activity, depending on the extracting solvent used. The numbers represent the assigned metabolites that are presented in the Figure 5 caption (1: quinic acid, 2: citric acid, 3: coumaroyl-caffeoylquinic acid isomer, 4: protocatechuic acid, 5: neochlorogenic acid, 6: chlorogenic acid, 7: kaempferol hexoside, 8: coumaroylquinic acid, 9: rutin, 10: quercetin hexoside, 11: quercetin malonylhexoside, 12: kaempferol rutinoside, 13: isorhamnetin rutoside, 14: isorhamnetin pentoside, 15: dicaffeoylquinic acid, 16: isorhamnetin hexoside, 17: kaempferol derivative, 18: naringenin).

Careful qualitative comparative analysis of the three extracts discussed above led to the preparation of Figure 6, presented below, which, while analyzing the peak areas of the selected metabolites, also visualized the differences in the fingerprint induced by differently polar solvents.

Based on the comparative data presented in Figure 6, it is evident that the presence of water strongly influenced the chemical fingerprint of the extracts. This effect is particularly clear in the lower panel, where the comparison between the UAE EtOH (8) and UAE EtOH–H_2_O mixture (1:1, *v*/*v*) (9) extracts demonstrates a high degree of similarity in compound composition, indicating that even moderate water content results in closely aligned extraction profiles. In contrast, the upper panel, which compares the UAE EtOH (8) extract with the UAE H_2_O (7) extract, highlights the substantial impact of water on the extraction process. Compounds numbered **14**, **18**, **16**, **15**, and **10**, described in Figure 6, are present at markedly higher levels in the water-free extract compared to Compounds **1**–**8**, emphasizing that these constituents are more efficiently extracted in the absence of water. Consequently, Compounds **14**, **15**, **16**, and **18** may play a predominant role in shaping the extracts’ anti-inflammatory potential, as their abundance is significantly higher in the EtOH sample (see also Figure S7 in the Supplementary File). These differences suggest that the presence and relative amounts of these key compounds are critical factors that differentiate the extracts in terms of their anti-inflammatory activity. According to the results shown in Figure S8, the ethanolic extracts exhibit the highest naringenin concentrations. Notably, its presence in samples obtained via maceration (SM) surpasses that extracted using ultrasound-assisted extraction (UAE) and indicates a more substantial macerate capacity. The obtained data suggest that naringenin may play a pivotal role in modulating the anti-inflammatory activity of the elderberry flower.

### 2.5. Cytotoxic Activity Assessment

**Figure 7:**
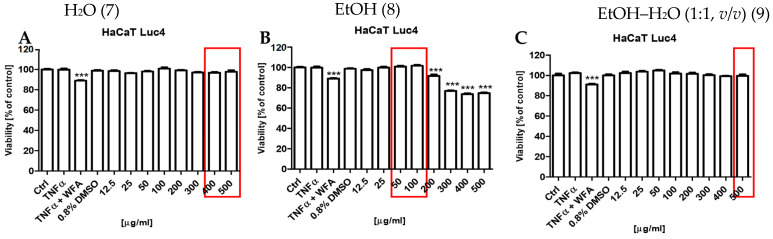
Effects of *S. nigra* flower extracts on cell viability: (**A**): H_2_O (7) extract, (**B**): EtOH (8) extract, (**C**): EtOH–H_2_O (1:1, *v*/*v*) (9) extract, at the concentrations of 12.5–500 µg/mL. The data are presented as the mean ± standard deviation (±SD) of the mean. *** *p* < 0.001 by one-way ANOVA, Tukey’s post hoc testing. Red frames—maximum non-toxic concentrations with anti-inflammatory potential of *S. nigra* flower extracts.

The potential cytotoxic effects of the *S. nigra* flower extracts (7, 8, and 9) on the human keratinocytes were assessed using the resazurin-based cell viability assay (Figure 7). The results demonstrated that only extract 8 exhibited statistically significant cytotoxic effects (*** *p* < 0.001) at concentrations ≥200 µg/mL (Figure 7B). In turn, extracts 7 (Figure 7A) and 9 (Figure 7C) did not reduce keratinocyte viability across the entire concentration range tested (12.5–500 µg/mL).

## 3. Discussion

The phytochemical profile of *S. nigra* is distinctly dichotomous, as proven by the scientific literature. The flowers serve as a rich source of phenolic acids (principally chlorogenic acid) and flavonols (dominated by rutin), whereas the fruits are characterized by an exceptionally high concentration of anthocyanins (cyanidin derivatives) [11]. The final chemical composition of any extract is highly contingent upon the morphological part used, the solvent system, and the specific extraction parameters employed, including the extracting solvent, as well as temperature and extraction duration, which will be further discussed.

Previous studies on *S. nigra* flower extracts have predominantly focused on single extraction techniques, most commonly maceration, infusion, or ultrasound-assisted extraction, often applied under a limited set of process conditions and evaluated mainly through total phenolic content or classical antioxidant and enzyme inhibition assays [20,21,22].

In contrast to other publications, the present study applies a systematic and comparative approach by evaluating three fundamentally different extraction techniques—ultrasound-assisted extraction, shaking maceration, and accelerated solvent extraction—across a broad range of extraction times, temperatures, and solvent polarities. This design enables the differentiation between solvent-driven and technique-dependent effects on extract composition, which has rarely been addressed in a unified framework.

The results of this study demonstrate that both the extraction technique and solvent composition markedly influenced the quantitative and qualitative profiles of elderflower extracts. Accelerated solvent extraction and ultrasound-assisted extraction generally yielded higher levels of major phenolic constituents, including rutin and chlorogenic acid, compared to shaking maceration, although solvent polarity played a decisive role in shaping the final metabolite composition.

The study by Uzlasir provides a comparative analysis of different solvents but applies a different technique, namely the preparation of an infusion [13]. In their studies, the highest rutin concentration (128.12 mg/L) was obtained with a 30 min methanolic extraction. For aqueous extractions, the duration effect was temperature-dependent; at 85 °C, extending the infusion from 5 to 30 min increased the rutin yield from 29.58 mg/L to 38.43 mg/L. According to their study, which was also the case in the protocols described herein, the most critical finding regarding rutin is its significant thermal degradation at elevated temperatures. In the presented studies, the longest extraction cycles did not yield the highest rutin recovery from the plant matrix. For Uzlasir, it was reported that while a 5 min infusion in water at 100 °C yielded 47.37 mg/L of rutin, extending the infusion time to 30 min at the same temperature caused a dramatic decrease in its concentration to just 8.27 mg/L. This indicates the severe thermal lability of the glycosidic bond or the aglycone itself under prolonged high-heat aqueous conditions. Ferreira-Santos, who optimized for total phenolic content, reported a high concentration of rutin (1887.7 µg/g of flower) in their 90 °C aqueous extract, suggesting that this temperature may represent a more favorable balance between extraction efficiency and thermal stability compared to 100 °C [9]. These assumptions were confirmed by a study on its stability during gastrointestinal digestion, which was the focus of Has’s investigation of elderberry fruit extract [12]. The initial concentration of 7.9 µg/mg decreased to 6.30 µg/mg in the simulated gastric fluid (SGF) and further to 5.42 µg/mg in the simulated intestinal fluid (SIF). This corresponds to a final bioaccessibility of approximately 60.6% for the flavonol class, indicating moderate stability.

Temperature had a pronounced effect on rutin extraction efficiency in our study as well, with elevated temperatures generally enhancing its recovery. In particular, extraction techniques operating at higher temperatures, such as accelerated solvent extraction, resulted in increased rutin yields compared to low-temperature approaches, indicating improved mass transfer and solvent penetration. However, the highest rutin contents were observed under moderate-to-high temperature conditions, combined with appropriate solvent polarity, suggesting that temperature alone was not the sole determining factor, in line with previously published reports on the impact of elevated temperature on the compound’s stability. Our results indicate that rutin extraction is dependent on both the effects triggered by thermal effects and solvent composition, rather than solely by temperature.

The scientific literature demonstrates that the optimal conditions for extracting rutin and chlorogenic acid from *S. nigra* are mutually exclusive. The extraction of chlorogenic acid is maximized by aggressive thermal treatment (100 °C, 30 min) in an aqueous medium. Conversely, rutin is thermally labile under these same conditions, and its recovery is compromised by prolonged exposure to high temperatures.

The case of chlorogenic acid recovery was described by Uzlasir, who reported the highest concentration for 3-caffeoylquinic acid (388.75 mg/L) in an aqueous infusion conducted at 100 °C for 30 min among all infusions tested [13]. This represents a substantial increase from the 329.76 mg/L obtained after only 5 min at the same temperature. This positive correlation is further supported by Ferreira-Santos, who selected their 90 °C extract for detailed analysis precisely because it exhibited the highest total phenolic content and antioxidant activity, a profile dominated by a total of 9146.5 µg/g of caffeoylquinic acid isomers [9]. Also, in the case of this study, extracts No. 25 and 9 (ASE at 100 °C for 5 min and UAE for 20 min, both with ethanol–water mixture (1:1, *v*/*v*), respectively), with an elevated temperature in the case of ASE and a long extraction time, were found to be rich in chlorogenic acid. However, unlike previous studies that indicated aqueous extraction at high temperatures as the most effective method for chlorogenic acid recovery, Uzlasir’s study found that ethanol–water mixture (1:1, *v*/*v*) was a more efficient solvent [13].

Chemometric analyses provided particularly informative insights into the impact of extraction conditions on the composition of *S. nigra* flower extracts. As demonstrated by the hierarchical clustering and heatmap analysis (Figure 3), solvent polarity was a major determinant of metabolite distribution, with water-based extracts being enriched in highly polar organic and hydroxycinnamic acids, including citric, quinic, chlorogenic, and neochlorogenic acids. In contrast, extracts obtained with high ethanol concentrations showed increased levels of less polar metabolites, notably flavonoid glycosides such as quercetin, isorhamnetin, and kaempferol derivatives, as well as dicaffeoylquinic acids. The PLS-DA model (Figure 4) further confirmed this solvent-driven separation, identifying hydroxycinnamic acids as markers of aqueous extracts, whereas flavonoid glycosides and dicaffeoylquinic acids were the most influential variables associated with ethanol-based extractions. Together, these results highlight that solvent selection, in combination with the extraction technique, plays a pivotal role in shaping the qualitative dominance and relative abundance of individual metabolite classes in elderflower extracts.

While most available reports investigate plant material originating from a single geographical location, the present work compares elderflower samples from Poland and Ukraine under identical extraction conditions, providing insight into the reproducibility of optimized extraction strategies and the influence of raw material origin on phenolic composition [20,23].

Quantitative analysis demonstrated that the levels of rutin and chlorogenic acid in Ukrainian elderflower extracts were consistently lower than those observed in Polish extracts, despite identical extraction conditions. These differences indicate that the geographical origin of elderflowers influences the accumulation of key phenolic compounds. The consistently lower levels of rutin and chlorogenic acid in Ukrainian material suggest that environmental or genetic factors contribute to the observed phytochemical variability.

Overall, these findings highlight the combined impact of extraction strategy and plant provenance on the chemical characteristics of *S. nigra* flower extracts.

Chemical characterization of *S. nigra* flower extracts usually involves the identification of a restricted number of major phenolic constituents and simple correlations with biological activity [23,24]. High-resolution HPLC-ESI-QTOF-MS/MS analysis combined with chemometric tools, including hierarchical clustering and PLS-DA, allowed for the identification of approximately twenty metabolites in this study and the discrimination of extraction-specific chemical fingerprints. Importantly, this multivariate approach revealed naringenin as a key contributor to the anti-inflammatory activity of selected extracts, whereas previous studies have mainly emphasized rutin and chlorogenic acid as principal bioactive markers [22,23].

The literature reports a high abundance of caffeoylquinic acid derivatives in elderberry flowers and fruits. Chlorogenic acid (5-*O*-caffeoylquinic acid) and its isomers, 3-*O*-caffeoylquinic acid (neochlorogenic acid) and 4-*O*-caffeoylquinic acid (cryptochlorogenic acid), are consistently identified as the principal phenolic constituents in floral preparations across aqueous, methanolic, and ethanolic extraction systems. Their prevalence is a defining characteristic of the elderflower chemical signature. Ferreira, Silva and Nunes identified 3-*O*-caffeoylquinic acid (neochlorogenic acid), 4-*O*-caffeoylquinic acid (cryptochlorogenic acid), and 5-*O*-caffeoylquinic acid (chlorogenic acid) as key cinnamic acids present in the plant [11]. Also, the authors underline an abundant presence of coumaroylquinic acid in various isomeric forms (3-*O-p*-, 4-O-p-, and 5-*O-p*-coumaroylquinic acid); coumaroyl–caffeoylquinic acid isomers, like the abovementioned 3-*O*-*p*-coumaroyl-4-O-caffeoylquinic acid; and feruloylquinic acid in both flowers and fruits [9,12,13].

Previously, the presence of quinic and citric acids was proven by Ferreira, Qazimi and Veberic, with the “Haschberg” cultivar being particularly rich in these components [10,11,14].

The flavonoid profile of *S. nigra* is markedly different between its morphological parts, with flavonols dominating in the flowers and anthocyanins in the fruits. Among flavonols, rutin (quercetin 3-*O*-rutinoside) is one of the most prominent and consistently reported flavonoids in *S. nigra*. Multiple studies have identified it as a major compound in berries and flowers, including the works of Veberic; Has; Uzlasir; Ferreira, Silva and Nunes; and Qazimi [10,11,12,13,14]. Similar to quercetin 3-*D*-glucoside, kaempferol-3-*O*-rutinoside, isorhamnetin 3-*O*-rutinoside, kaempferol 3-*O*-glucoside, and isorhamnetin-3-*O*-glucoside were validated by Ferreira, Silva and Nunes, as well as by Has, Uzlasir and Qazimi [11,12,13,14]. Our assignment of quercetin malonylhexoside is supported by the findings of Qazimi, who detected quercetin malonyl-diglucoside, indicating that malonylated quercetin glycosides are indeed constituents of *S. nigra* [14]. Naringenin—a commonly spread flavanone—has been identified in elderflower extracts as a dominating component of unpolar extracts [11,14].

Although the qualitative phytochemical profile observed in this study agrees with previous reports on *S. nigra* flowers, the present work extends existing knowledge by providing a systematic, solvent-dependent comparison supported by high-resolution mass spectrometry and chemometric analysis. This approach enabled the identification of extraction-selective enrichment patterns for major phenolic subclasses and revealed additional metabolites, including spermidine and pinellic acid, thereby broadening the reported chemical spectrum of elderflower extracts.

Finally, the biological activity of the extracts was assessed using a stably transfected HaCaT NF-κB luciferase reporter cell line, enabling direct evaluation of NF-κB pathway modulation in human keratinocytes. Such a mechanistically relevant inflammatory model has not previously been applied to *S. nigra* flower extracts, extending earlier studies that relied primarily on antioxidant assays or non-specific cell viability tests [21,25]. The integration of cell-based assays with metabolite fingerprinting and chemometric analysis enabled the identification of specific metabolites associated with extracts exhibiting enhanced anti-inflammatory activity. In this context, naringenin emerged as one of the compounds contributing to the differentiation of more active extracts, suggesting that it may participate—together with other co-occurring metabolites—in shaping the overall anti-inflammatory potential of the extracts.

An analysis of the scientific literature shows substantial evidence supporting the anti-inflammatory potential of this flavonoid, demonstrated across various models and conditions. In macrophage and ex vivo human whole-blood models, naringenin effectively inhibited the production of pro-inflammatory cytokines, including interleukin-1β, interleukin-6, interleukin-8, and tumoral necrosis factor-α, upon stimulation with lipopolysaccharides from pathogens such as *Aggregatibacter actinomycetemcomitans* and *Escherichia coli* [26]. In these studies, naringenin concentrations ranging from 10 to 50 μg/mL significantly reduced cytokine levels, indicating its potential therapeutic application in periodontal inflammation and other inflammatory diseases. Furthermore, naringenin’s anti-inflammatory efficacy extends to various in vivo models. It has been shown to attenuate inflammation in models of rheumatoid arthritis by modulating the NF-κB pathway and reducing oxidative stress [27]. Naringenin also exhibits protective effects in models of UVB-induced skin inflammation and other inflammatory conditions by inhibiting leukocyte recruitment and cytokine production [26,27].

The dosage and model specificity are crucial in naringenin’s application. For instance, in a collagen-induced arthritis model in rats, naringenin effectively reduced joint inflammation and cytokine production, highlighting its role in modulating immune responses in autoimmune diseases [28]. These findings suggest that naringenin, through its multi-targeted anti-inflammatory and antioxidant mechanisms, holds promise as a therapeutic agent for managing chronic inflammatory conditions.

The anti-inflammatory activity of the EtOH extract reported in this study towards the HaCaT NF-κB Luc reporter cell line proved that elderberry flower may affect inflammatory conditions in the skin by mitigating the activation of the NF-κB pathway at non-cytotoxic concentrations. Since the investigation was performed on HaCaT keratinocytes, which serve as a well-established in vitro model for the human epidermis, the most direct applications for this *S. nigra* extract lie in dermatological and cosmetic formulations. These findings suggest that the extract could be a valuable ingredient in treatments for various NF-κB-driven skin disorders. For instance, in atopic dermatitis, where NF-κB is a key mediator of pathogenesis, its inhibition could lead to a significant reduction in clinical symptoms such as pruritus, erythema, and inflammation. Similarly, in psoriasis, which is characterized by excessive NF-κB activation, the extract may serve as a component in soothing and modulating preparations while also offering benefits for managing eczema and general skin irritation. Furthermore, its potential to reduce inflammation by inhibiting NF-κB, which regulates pro-inflammatory cytokines such as IL-6 and IL-8, makes it a promising candidate for acne treatment. Beyond treating specific pathologies, the extract’s regulatory effect on NF-κB signaling positions it as a potent agent in anti-aging applications, as this pathway is involved in cellular senescence and chronic “inflammaging,” which contribute to the formation of wrinkles and secondary hyperpigmentation. The dual anti-inflammatory properties and potential have been previously proven by the scientific literature; antioxidants can, therefore, support skin regeneration, enhance barrier function, and reduce oxidative stress. Finally, these activities are also highly beneficial for wound healing; by inhibiting excessive inflammation, the extract can foster an optimal environment for tissue repair, potentially leading to faster healing, reduced scar formation, and more effective skin remodeling.

## 4. Materials and Methods

### 4.1. Chemicals and Reagents

The following reagents were used: double-distilled water for extraction; absolute ethyl alcohol (99.8%) (POCH, Gliwice, Poland); methanol (POCH, Gliwice, Poland); HPLC-grade acetonitrile (JT Baker, Philipsburg, NJ, USA); HPLC-grade formic acid (JT Baker, USA); HPLC-grade water (JT Baker, Philipsburg, NJ, USA). The HPLC-MS analysis was performed using mass-spectrometry-purity water, formic acid and acetonitrile, all of which were ordered from JT Baker.

Standards used for extract identification and calibration curve construction were purchased from Sigma-Aldrich (St. Louis, MO, USA) in a purity exceeding 95%: rutin trihydrate, quercetin, caffeic acid, and chlorogenic acid.

### 4.2. Plant Material

The plant material used in this study consisted of black elderflowers (*Sambucus nigra* L., *Sambuci flos*), produced by Flos (Paslek, Poland, nr of series: 5909990628223), and purchased in dried form from a local shop in Lublin in autumn 2024. For comparative analysis, dried black elderflowers from the manufacturer Lictravy (Zhytomyr, Ukraine nr of series: 4823012801153), purchased in a pharmacy in Ukraine, were used.

### 4.3. Extraction

The primary objective of this work was to obtain a comprehensive comparative evaluation of fundamentally different extraction techniques (ultrasound-assisted extraction, shaking maceration, and accelerated solvent extraction) and solvent systems in order to capture a wide range of chemical fingerprints relevant for subsequent chemometric and biological analyses. For this purpose, plant material extraction was carried out using three techniques: maceration with shaking, ultrasound-assisted extraction (UAE), and accelerated solvent extraction (ASE). These three extraction methods represent commonly applied approaches used in phytochemical and natural product research and show differences in extraction efficiency, intensity, and technological complexity. For shaking and ultrasound-assisted extraction, four extraction times were applied (5, 10, 20, and 30 min). In the case of ASE, three extraction times (5, 10, and 15 min) and three temperatures (60 °C, 80 °C, and 100 °C) were tested. Each method was performed with three different solvents: absolute ethanol (99.8%), a 1:1 (*v*/*v*) ethanol–water mixture, and water. Various extraction times were selected to cover short to longer extraction durations commonly applied in laboratory-scale studies, allowing us to assess the extraction kinetics. The range of temperatures applied in ASE enabled us to elevate temperature-dependent extraction efficiency and to avoid excessive thermal degradation of compounds characteristic of the investigated plant material. The selection of solvents was intended to span a broad polarity range and to reflect solvent systems frequently employed for the extraction of phenolic compounds from plant materials. Shaking and ultrasound-assisted extraction were conducted in triplicate for each solvent and extraction time due to higher experimental variability, while ASE, as a highly automated and reproducible technique, was performed in duplicate for each solvent, extraction time, and temperature. Plant material of Ukrainian origin was extracted using three selected methods, namely ASE (5 min at 100 °C), UAE (20 min), and shaking maceration (10 min), in triplicate. All of them used ethanol–water mixture (1:1, *v*/*v*) as an extracting solvent. These extraction methods and the solvent were found to recover the highest loads of rutin and chlorogenic acid from black elderberry flowers and to induce the strongest radical scavenging activity in the flower samples of Polish origin; therefore, these conditions were selected for the Ukrainian species. The list of obtained extracts is presented in Table 2 of the Section 2.

#### 4.3.1. Shaking and Ultrasound Extraction

One gram of plant material was weighed into Falcon tubes, and 5 mL of the appropriate solvent (water, ethanol, or ethanol–water mixture (1:1, *v*/*v*)) was added, corresponding to a drug-to-solvent ratio of 1:5 (*w*/*v*). This procedure yielded a total of 36 extracts, obtained at four extraction times (5, 10, 20, and 30 min) in an ultrasonic bath. Each extraction was performed in triplicate for every time point. Ultrasound-assisted extraction (UAE) was performed using an ultrasonic bath (Emmi 100HC, EMAG AG, Mörfelden-Walldorf, Germany) operating at a frequency of 40 kHz with a maximum ultrasonic power of 360 W. The extraction was carried out at room temperature. Shaking-assisted extraction was achieved using a laboratory platform shaker (INFORS AG, Bottmingen, Switzerland) operated at the maximum shaking speed (400 rpm) at room temperature. Following the extraction procedure, all samples were centrifuged for 10 min to separate the liquid phase. The supernatant was collected and filtered through a syringe filter into Eppendorf tubes. The prepared samples were dried in a vacuum concentrator at 45 °C for 7 h. After complete solvent evaporation, the dried residues were weighed and used for further analysis. The same procedure was applied for the shaking method, with the ultrasonic bath replaced by a laboratory shaker.

#### 4.3.2. Accelerated Solvent Extraction (ASE)

Plant material (2 g) was placed in a 34 mL extraction cell fitted with a filter and subjected to accelerated solvent extraction (ASE 100, Dionex, Sunnyvale, CA, USA), corresponding to a drug-to-solvent ratio of approximately 1:17 (*w*/*v*), using ethanol, ethanol–water mixture (1:1, *v*/*v*), and distilled water as solvents. Extraction parameters were as follows: time—5, 10, and 15 min; temperature—60, 80, and 100 °C; pressure—100 bar. Each extraction was performed in duplicate for every time–temperature combination. This approach was undertaken in view of the technical and material constraints; however, after checking that the duplicated extracts were very close to each other in terms of dried residue weight and qualitative and quantitative fingerprint. The obtained solutions were transferred to 250 mL round-bottom flasks, and the excess solvent was removed under reduced pressure using a rotary evaporator at 40 °C. For aqueous extracts, solvent removal was performed using an Eppendorf Concentrator Plus centrifuge (Eppendorf, Hamburg, Germany). The dried residues were subsequently dissolved in a small amount of the extraction solvent, transferred to 1.5 mL Eppendorf tubes, and dried again in a vacuum concentrator at 45 °C. Finally, the dried extracts were weighed and stored for further analysis at 4 °C—no longer than for the following 2 weeks.

### 4.4. Fingerprinting of Extracts by HPLC-PDA and HPLC-MS

Dried extracts were prepared for HPLC-PDA and HPLC-MS analysis by dissolving them in an appropriate solvent (depending on the extract type) to a final concentration of 5 mg/mL. Each extract from every extraction method was analyzed. The resulting solutions were vortex-mixed for 10 min and subsequently filtered through 0.1 µm syringe filters (Filterbio, Nantong, China) into 2 mL vials. Quantitative analysis was performed on a Prominence-i LC-2030 3D HPLC system (Shimadzu, Kyoto, Japan) equipped with the PDA detector, degasser, autosampler, thermostated column chamber and quaternary pump. The qualitative HPLC-MS fingerprinting of the selected extracts was achieved using a platform from Agilent Technologies (Santa Clara, CA, USA) that was composed of a degasser (G1322A), a binary pump (G1312C), a thermostated column chamber, a PDA detector (G1315D) and an ESI-QTOF-MS/MS detector (G6530B). Both instruments were equipped with the same chromatographic column, namely a Zorbax Eclipse Plus RP-18 column (Agilent Technologies, Santa Clara, CA, USA), and were operated using the same gradient of acetonitrile with 0.1% formic acid (channel B) in water with 0.1% formic acid (channel A), namely: 1% B at 0 min; increased to 15% at 5 min, 25% at 15 min, 35% at 25 min, and 95% at 30–35 min; and then again by 1% at 36–45 min. The flow rate was 0.2 mL/min, the injection volume was 10 µL, and the total run time was 45 min. The injections on the HPLC-PDA chromatograph were performed in triplicate. For the HPLC-MS-based quality assessment, the following mass spectrometer settings were used: collision energy of 3000 V, gas and sheath gas temperatures of 325 and 275 °C, gas flow of 12 L/min, fragmentor energy of 110 V, collision energy values of 10 and 20 V, and skimmer voltage of 65 V. Instrument control and data acquisition for the quantitative assessment were performed using the LabSolutions LC software, whereas HPLC-MS-based fingerprinting was performed using Agilent MassHunter Workstation (version B.12.00).

For the quantitative analysis, the solutions of two reference compounds were prepared (see Figure S7 and Table S2 in the Supplementary File). The standards of rutin and chlorogenic acid, with a purity exceeding 95%, were purchased from Sigma Aldrich (St. Louis, MO, USA). For quantification, five differently concentrated working solutions of each standard were prepared in methanol (1.0, 0.75, 0.25, 0.125, and 0.06 mg/mL) by diluting the 5 mg/mL stock solution. Based on the calibration curves and regression equations obtained for rutin and chlorogenic acid standards, quantitative analysis of *S. nigra* flower extracts was performed. The relative contents of these compounds were calculated from the HPLC peak areas of the analyzed extracts using Microsoft Excel. In addition, the linearity range was determined for the aforementioned compounds. First, calibration curves for rutin and chlorogenic acid were constructed from five concentration levels in triplicate, as described in the methodology section. The curves exhibited good linearity within the tested range, 1 mg/mL to 0.06 mg/mL, with correlation coefficients of R^2^ = 0.9971 for rutin and R^2^ = 0.9934 for chlorogenic acid (Figure S7). The linear regression equations were y = 53,739,438.14x + 2,255,289.04 for rutin and y = 77,770,269.31x + 1,037,401.70 for chlorogenic acid. These results enabled direct calculations of the rutin and chlorogenic acid content in the studied samples. Moreover, the LOD and LOQ parameters for the detection of the two selected natural products were calculated. The LOD for rutin was 1.35 µg/mL, and for chlorogenic acid, it was 1.09 µg/mL, whereas their LOQ values were 4.44 and 3.62 mg/mL, respectively, corresponding to 3.3 times the LOD.

### 4.5. Chemometric Studies and Statistical Analysis

The statistical analysis of the extracts’ composition was performed by HPLC-MS, and the obtained data were further processed to deliver information about the existing correlations between the extracting solvent and the composition of the extract.

For the purposes of this analysis, all obtained extracts were analyzed using a UHPLC-QTOF-MS platform from Waters (Milford, MA, USA). The analysis was performed using a Waters Acquity Premier System (UPLC), coupled with an Xevo G3 high-resolution QTof-MS from Waters. Separation was achieved using a RRHD Zorbax C18 column (2.1 × 100 mm, 1.8 μm particle size) by Agilent Technologies (Santa Clara, CA, USA). The mobile phases were 0.1% formic acid in water (A) and 0.1% formic acid in acetonitrile (B), at a flow rate of 0.45 mL min^−1^ and an injection volume of 2 μL. The column oven was set at 50° C, and the LC gradient consisted of the following linear steps as min/B%: 0/1%, 2/25%, 4/35%, 6/35%, 10/95%, 13/95%, 13.1/1%, 17/1%. The autosampler temperature was maintained at 10 °C. Ionization was achieved by electrospray ionization (ESI) in negative mode, with a mass range of *m*/*z* 50–1200. Using the MS^E^ mode, a low energy MS (Collision Energy: 6 V) and a high energy MS (collision energy: 15–35 V) were measured in the same run. The capillary voltage was set at 2.5 kV with the sample cone voltage at 30 V, with a source temperature of 100 °C and a desolvation temperature of 250 °C. Nitrogen was used as a collision gas. The cone gas flow rate was set at 50 L h^−1^ and the desolvation gas at 1000 L h^−1^. Mass accuracy was confirmed prior to the run using sodium formate signals and during the runs using a lock mass correction.

All injections were performed in triplicate, and the analysis was run in DIA mode.

Chemometric processing of the UHPLC–MS data was performed on the series of injections described in this study. Raw chromatographic files were first processed using MZmine (ver. 4.7.8) [23] for peak detection, deconvolution, alignment, and relative peak intensity estimation. The workflow included mass detection, chromatogram building, deconvolution, and peak alignment across all samples to generate a comprehensive feature table. The resulting peak intensity matrix was exported from MZmine and subsequently uploaded to MetaboAnalyst 5.0 [29] for multivariate statistical analysis. Prior to modelling, the dataset underwent quality filtering. Normalization was performed using cube root transformation. Multivariate analyses, including hierarchical clustering and PLS-DA, were conducted using MetaboAnalyst’s built-in algorithms. Model validity and robustness were evaluated using cross-validation metrics (accuracy, R^2^, and Q^2^), permutation testing, and assessment of explained variance by individual components. VIP scores and loading plots were used to identify the metabolites that contributed most strongly to group separation. Heatmaps were generated to visualize relative metabolite abundance patterns across extraction methods and solvent conditions. Heatmap visualization, hierarchical clustering using Ward’s linkage with Euclidean distance, and partial least squares discriminant analysis (PLS-DA) were performed using the MetaboAnalyst platform, 6.0.

### 4.6. Cell Culture

For the biological assays, a subset of three extracts was selected based on their favorable quantitative extraction yields, high overall metabolite abundance, and distinct chemical fingerprints resulting from the use of three different extraction solvents (absolute ethanol, ethanol–water mixture 1:1, *v*/*v*, and water), while keeping the extraction technique and duration constant. This selection strategy was adopted to enable a comparative assessment of how solvent-driven differences in metabolite composition influence the overall anti-inflammatory activity of elderflower extracts. To study the impact of a solvent on the bioactivity of the investigated extracts, the cytotoxic and anti-inflammatory assays were performed using HaCaT NF-κB Luc reporter cells on three UAE extracts obtained with water, ethanol–water mixture (1:1, *v*/*v*), and absolute ethanol prepared within 20 min [30]. The cells were maintained in Dulbecco’s Modified Eagle Medium (DMEM) (Gibco; New York, NY, USA) supplemented with 10% fetal bovine serum (FBS) and sodium pyruvate under a humidified atmosphere containing 5% CO_2_ at 37 °C. For the cytotoxicity and anti-inflammatory assays, medium supplemented with 2% FBS was used for the treatment with *S. nigra* flower extracts. Puromycin (1 µg/mL) (Gibco, Thermo Fisher Scientific; Waltham, MA, USA) was used for clone selection.

### 4.7. Cell Viability Assay

The cytotoxic potential of *S. nigra* flower extracts was evaluated using the resazurin reduction assay. A total of 17.000 HaCaT NF-κB Luc reporter cells were seeded into 96-well culture plates and incubated overnight. On the following day, the culture medium was replaced with medium containing 2% FBS to induce serum starvation. After 24 h, cells were treated with the extracts at concentrations ranging from 12.5 to 500 µg/mL for 4 h. Subsequently, the samples were removed, and cells were incubated with 0.015 mg/mL resazurin (Sigma-Aldrich; St. Louis, MO, USA) for 2 h before fluorescence measurement. Cell viability was quantified by resazurin-based fluorometric analysis, with fluorescence intensity measured using a microplate reader (Tecan Spark, Tecan Group Ltd.; Männedorf, Switzerland) at excitation/emission wavelengths of 560/590 nm. All experiments were performed in triplicate.

### 4.8. Luciferase Reporter Assay

To evaluate the potential anti-inflammatory activity of *S. nigra* flower extracts, the NF-κB signaling pathway was activated in HaCaT NF-κB Luc reporter cells by stimulation with tumor necrosis factor alpha (TNF-α, 0.75 ng/mL final concentration) (PeproTech; Cranbury, NJ, USA). The stimulated cells were simultaneously treated with various concentrations of the extracts (12.5–500 µg/mL) for 4 h. Withaferin A (WFA), a known inhibitor of NF-κB signaling through direct disruption of p65 subunit dimerization, was employed as a positive control for NF-κB inhibition [31]. Following treatment, cells were washed with ice-cold 1× phosphate-buffered saline (PBS) and extracted with Lysis buffer (250 mM Tris-HCl, pH 7.5, 2% Triton X-100) for 5 min at RT. Subsequently, 50 μL of lysate was mixed with 150 μL of Luciferase Assay buffer (25 mM glycylglycine, pH 7.8, 5 mM ATP, 15 mM MgSO_4_) and injected with 50 μL of Luciferase Substrate buffer (20 mM glycylglycine, pH 7.8, 28.5 µM (4S) 2-(6-hydroxy-1,3-benzothiazol-2-yl)-4,5-dihydrothiazole-4-carboxylic acid). Luminescence intensity was quantified using a multimode microplate reader (Tecan Spark, Tecan Group Ltd.; Männedorf, Switzerland).

### 4.9. Statistical Analysis

One-way analysis of variance (ANOVA) and Tukey’s post-hoc testing were used to evaluate the differences between the indicated values from the cell viability and luciferase reporter assay using GraphPad Prism 5.0 (GraphPad Software Inc.; San Diego, CA, USA). Results were statistically significant if *p* < 0.05 (* *p* < 0.05, ** *p* < 0.01, *** *p* < 0.001).

## 5. Conclusions

The results of this study highlight a comprehensive profile and the presence of a rich array of polyphenolic compounds, including flavonoids such as rutin and naringenin, and phenolic acids such as chlorogenic acid, which was confirmed in HPLC-ESI-QTOF-MS/MS fingerprinting. These compounds were found to be abundant in the extracts, contributing to their potent anti-inflammatory properties. It is worth noting that the extraction conditions, particularly solvent selection, played a key role in determining the phenolic content and bioactivity of the extracts. The quantitative fingerprinting of extracts in terms of the rutin and chlorogenic acid content, which were the most abundant components found in the elderberry extracts, indicates that hydroethanolic mixtures (EtOH–H_2_O (1:1, *v*/*v*) extracts) proved to be the most effective, maximizing the recovery of these two key phenolic compounds. The extract obtained by ultrasound-assisted extraction for 20 min in ethanol–water mixture (1:1, *v*/*v*) was selected as the richest in rutin and chlorogenic acid, with measured percentage contents of 4.87% and 8.22% (n = 3), respectively. Interestingly, naringenin was abundant in the ethanolic extracts, highlighting the importance of solvent selection in optimizing the yield of individual bioactive compounds. This study also highlighted the impact of extraction techniques, with ultrasound-assisted extraction (UAE) proving superior in preserving bioactive components. The statistical analysis of the recorded mass chromatograms identified naringenin as one of the metabolites associated with the differentiation of extracts exhibiting higher anti-inflammatory activity. The scientific literature has proven naringenin’s remarkable ability to inhibit proinflammatory cytokines and modulate oxidative stress pathways, making it a promising therapeutic agent in the treatment of inflammation. The observed inhibition of NF-κB activity should be interpreted as the combined effect of the complex metabolite composition of the extracts rather than being attributed to a single flavonoid. Due to the inhibition of NF-κB activation in skin keratinocytes at non-cytotoxic concentrations, the extract may be perceived as a candidate for application in dermatological and cosmetic formulations. Its activity suggests therapeutic utility in treating NF-κB-dependent skin disorders, such as atopic dermatitis, psoriasis, and acne. Furthermore, by modulating the “inflammaging” process and supporting tissue repair, the extract is a promising ingredient for anti-aging products and wound-healing preparations. Its anti-inflammatory properties make it a versatile ingredient for enhancing skin regeneration, strengthening the epidermal barrier, and reducing oxidative stress. The prevalence of naringenin in ethanolic extracts highlights the crucial role of extraction parameters in enhancing the therapeutic potential of elderflower extracts.

This study confirms the need for further research into the in vivo applications of elderflower extracts, utilizing predictive statistical models to further elucidate the mechanisms underlying their anti-inflammatory efficacy on the way towards the development of standardized phytopharmaceuticals that fully utilize the potential of elderflower. Multivariate analysis revealed distinct chemical fingerprints among the extracts and identified naringenin as one of the metabolites contributing to the differentiation of extracts exhibiting higher anti-inflammatory activity in the NF-κB reporter assay. However, the observed NF-κB inhibition should be interpreted as the result of the combined action of multiple metabolites rather than being attributed to a single compound. Overall, the integrative, analytical, and biological approach applied in this study provides valuable insights into how extraction parameters and plant provenance shape the anti-inflammatory potential of elderflower extracts and supports their further investigation for dermatological and cosmetic applications.

## Figures and Tables

**Table 1:** The results of quantitative analysis of rutin and chlorogenic acid in *S. nigra* flower extracts obtained by different extraction methods in the HPLC-PDA study in % content (±SD).

				Rutin			Chlorogenic Acid		
UAE									
Extract ID	Solvent	Time (min)	Temperature	Mean (%)	SD (%)	RSD (%)	Mean (%)	SD (%)	RSD (%)
1	EtOH-H_2_O	5	Room temp.	3.46	0.46	0.13	4.77	0.57	0.12
2	EtOH	5	Room temp.	−0.08	0.14	n/a	0.46	0.61	1.33
3	H_2_O	5	Room temp.	2.69	1.41	0.48	2.06	2.79	1.35
4	H_2_O	10	Room temp.	1.17	0.42	0.36	3.47	0.99	0.29
5	EtOH	10	Room temp.	0.61	0.91	1.50	0.64	0.89	1.38
6	EtOH-H_2_O	10	Room temp.	2.52	0.45	0.18	4.13	0.43	0.11
7	H_2_O	20	Room temp.	0.93	0.37	0.40	3.38	0.04	0.01
8	EtOH	20	Room temp.	0.80	0.34	0.43	0.13	0.34	2.49
9	EtOH-H_2_O	20	Room temp.	4.87	2.45	0.50	8.22	4.02	0.49
10	H_2_O	30	Room temp.	1.17	0.18	0.15	3.98	0.66	0.16
11	EtOH	30	Room temp.	1.76	0.62	0.35	1.26	1.41	1.11
12	EtOH-H_2_O	30	Room temp.	2.32	1.31	0.57	4.52	2.68	0.59
Shaking maceration									
13	H_2_O	5	Room temp.	−0.07	0.14	n/a	1.4	0.14	0.1
14	EtOH-H_2_O	5	Room temp.	2.76	0.03	0.01	3.99	0.29	0.07
15	EtOH	5	Room temp.	0.73	0.15	0.21	0.05	0.07	1.56
16	H_2_O	10	Room temp.	0.55	0.23	0.41	2.22	0.66	0.30
17	EtOH-H_2_O	10	Room temp.	3.20	0.37	0.11	4.71	0.50	0.11
18	EtOH	10	Room temp.	0.69	0.14	0.21	0.22	0.31	1.42
19	H_2_O	20	Room temp.	0.16	0.11	0.67	2.37	0.40	0.17
20	EtOH-H_2_O	20	Room temp.	1.30	0.07	0.05	3.84	0.21	0.05
21	EtOH	20	Room temp.	0.78	0.52	0.67	0.11	0.57	4.96
22	H_2_O	30	Room temp.	0.00	0.30	n/a	1.08	0.49	0.45
23	EtOH-H_2_O	30	Room temp.	2.55	0.58	0.23	4.56	0.99	0.22
24	EtOH	30	Room temp.	0.88	0.30	0.34	0.35	0.41	1.16
ASE									
25	EtOH-H_2_O	5 min	100 °C	2.73	0.08	0.03	5.01	0.53	0.11
26	EtOH-H_2_O	5 min	60 °C	2.34	0.30	0.13	3.86	0.54	0.14
27	EtOH-H_2_O	5 min	80 °C	2.53	0.35	0.14	4.31	0.72	0.17
28	EtOH-H_2_O	10 min	60 °C	0.81	1.58	1.97	1.11	3.08	2.77
29	EtOH-H_2_O	10 min	80 °C	2.05	1.98	0.96	−0.64	0.01	n/a
30	EtOH-H_2_O	10 min	100 °C	2.64	0.43	0.16	−0.68	0.00	0.00
31	EtOH-H_2_O	15 min	60 °C	1.93	0.72	0.37	2.90	3.18	1.10
32	EtOH-H_2_O	15 min	80 °C	2.03	0.16	0.08	3.74	0.35	0.09
33	EtOH-H_2_O	15 min	100 °C	1.00	1.53	1.53	4.64	1.67	0.36
34	EtOH	5 min	60 °C	1.85	0.14	0.08	2.10	1.18	0.56
35	EtOH	10 min	60 °C	1.92	0.21	0.11	1.57	0.50	0.32
36	EtOH	15 min	60 °C	2.39	0.65	0.27	2.11	1.09	0.52
37	EtOH	5 min	80 °C	1.81	0.13	0.07	1.76	0.30	0.17
38	EtOH	10 min	80 °C	1.97	0.15	0.08	1.75	0.29	0.17
39	EtOH	15 min	80 °C	1.55	0.27	0.18	1.71	0.13	0.08
40	EtOH	5 min	100 °C	1.98	0.26	0.13	2.08	0.26	0.12
41	EtOH	10 min	100 °C	2.54	0.68	0.27	2.61	0.81	0.31
42	EtOH	15 min	100 °C	3.25	1.60	0.49	2.82	0.84	0.30
43	H_2_O	5 min	100 °C	2.49	0.07	0.03	4.70	0.35	0.07
44	H_2_O	10 min	100 °C	1.43	0.45	0.32	4.08	0.53	0.13
45	H_2_O	15 min	100 °C	1.79	0.34	0.19	4.31	0.73	0.17
46	H_2_O	5 min	60 °C	1.30	0.07	0.05	3.84	0.21	0.05
47	H_2_O	10 min	60 °C	1.71	0.63	0.37	4.97	0.78	0.16
48	H_2_O	15 min	60 °C	0.83	0.36	0.43	3.44	0.71	0.21
49	H_2_O	5 min	80 °C	1.23	0.11	0.09	3.67	0.54	0.15
50	H_2_O	10 min	80 °C	1.41	0.26	0.18	3.94	0.83	0.21
51	H_2_O	15 min	80 °C	1.24	0.16	0.13	3.70	0.37	0.10
Ukr UAE	EtOH-H_2_O	20 min	Room temp.	2.93	0.65	0.22	5.00	1.25	0.25
Ukr Shak	EtOH-H_2_O	10 min	Room temp.	2.70	0.11	0.04	4.80	0.31	0.07
Ukr ASE	EtOH-H_2_O	5 min	100 °C	2.05	0.03	0.01	3.74	0.06	0.02

*Note:* Mean (%) represents the average percentage of rutin/chlorogenic acid in the extract obtained by the respective extraction method, calculated from three (UAE, shaking maceration) or two (ASE) independent replicates. SD—standard deviation; RSD (%)—relative standard deviation; n/a—not applicable. The number of replicates was n = 3 for UAE and shaking and n = 2 for ASE. UAE—ultrasonic-assisted extraction; ASE—accelerated extraction; EtOH—pure ethanol; H_2_O—water; EtOH-H_2_O—ethanol–water mixture (1:1, *v*/*v*); Ukr UAE, Ukr Shak, Ukr ASE—extracts prepared from Ukrainian raw material by the aforementioned methods.

**Table 2:** Tentative identification of the major compounds in the *S. nigra* flower extract by HPLC-ESI-QTOF-MS/MS analysis in negative ionization mode (* compared with a standard; DBE—number of double bonds and rings; Δ ppm—error of *m*/*z* measurement, the letters placed at the numbers of ions indicate the occurrence of the isomers with the same *m/z*).

No	Rt (min)	Theoretical *m*/*z*	Experimental *m*/*z*	Formula	Δ [ppm]	DBE	MS/MS Fragments	Annotation	References
1	1.92	191.0561	191.0541	C_7_H_12_O_6_	10.47	2	85, 111	Quinic acid	[9]
2	2.26	191.0197	191.0200	C_6_H_8_O_7_	1.43	3	111, 96, 87	Citric acid	[10,11]
3	11.01	153.0193	153.0198	C_7_H_6_O_4_	−3.04	5	109	Protocatechuic acid	[12]
4A	11.18	353.0878	353.0878	C_16_H_18_O_9_	0.02	8	191, 179, 135	Chlorogenic acid isomer 1	[9,13]
4B	11.68	353.0878	353.0876	C_16_H_18_O_9_	0.58	8	191, 179, 135	Chlorogenic acid isomer 2	[9,13]
4C	13.35	353.0878	353.0858	C_16_H_18_O_9_	5.66	8	191, 179, 173, 135	3-Caffeoylquinic acid (Chlorogenic acid) *	[9,13]
5	15.27	337.0929	337.0918	C_16_H_18_O_8_	3.23	8	191, 173	Coumaroylquinic acid	[9,13]
6	17.85	609.1461	609.1447	C_27_H_30_O_16_	2.31	13	301	Rutin (quercetin-3-*O*-rutinoside) *	[9,13]
7	18.60	377.1758	377.1750	C_24_H_26_O_4_	2.2	12	331, 179	3′,5′-Diprenylliquiritigenin	Metlin database
8	19.35	463.0882	463.0882	C_21_H_20_O_12_	0	12	300, 255	Quercetin 3-*D*-glucoside	[14]
9	19.60	593.1512	593.1514	C_27_H_30_O_15_	−0.35	13	357, 327, 285	Kaempferol-3-*O*-rutinoside	[13]
10	19.94	623.1618	623.1615	C_28_H_32_O_16_	0.41	13	315, 300	Isorhamnetin 3-*O*-rutinoside	[9,13]
11	19.94	549.0886	549.0888	C_24_H_22_O_15_	−0.38	14	300, 505	Quercetin malonylhexoside	[14]
12	20.60	447.0933	447.0937	C_21_H_20_O_11_	−0.03	12	284	Kaempferol 3-*O*-glucoside	[15]
13A	20.85	515.1195	515.1186	C_25_H_24_O_12_	1.74	14	353	Dicaffeoylquinic acid isomer 1	[9,13]
14	21.10	477.1038	447.1045	C_16_H_18_O_9_	−1.36	12	314	Isorhamnetin-3-*O*-glucoside	[16]
13B	21.77	515.1195	515.1207	C_25_H_24_O_12_	−2.33	14	353	Dicaffeoylquinic acid isomer 2	[9,13]
15A	23.44	538.2559	538.2556	C_29_H_36_N_3_O_7_	0.51	13	388	Spermidine isomer 1	[17]
16A	23.52	499.1246	499.1247	C_25_H_24_O_11_	−0.23	14	353, 337, 191	3-*O*-*p*-coumaroyl-4-*O*-caffeoylquinic acid isomer 1	[14]
16B	24.52	499.1246	499.1249	C_25_H_24_O_11_	−0.63	14	353, 337, 191	3-*O*-p-coumaroyl-4-*O*-caffeoylquinic acid isomer 2	[14]
15B	24.69	538.2559	538.2578	C_29_H_36_N_3_O_7_	0.51	13	388	Spermidine isomer 2	[17]
17	30.69	327.2177	327.2177	C_18_H_32_O_5_	−0.01	3	229, 211, 171	Linoleic acid derivative	[18]
18	31.28	271.0612	271.0602	C_15_H_12_O_5_	3.66	10	151	Naringenin *	[14]
19	32.03	285.0405	285.0411	C_15_H_10_O_6_	−2.23	11	-	Kaempferol	[9]
20	32.11	329.2333	329.2341	C_18_H_34_O_5_	−2.28	2	229, 211, 139	Pinellic acid	[19]

## Data Availability

The data are present in the manuscript body and in the Supplementary File published with the manuscript.

## Supplementary Materials

The following supporting information can be downloaded at https://www.mdpi.com/article/10.3390/molecules31030561/s1: Table S1: MS/MS chromatograms with fragmentation of identified compounds. Figure S1: Base peak chromatogram (BPC) obtained in the negative ionization mode (HPLC-ESI-QTOF-MS/MS) from the elderberry flower (Ukrainian origin) extracted in ultrasounds for 20 min using EtOH-H_2_O mixture (1:1, *v*/*v*). The peaks are numbered according to the compounds listed in Table 2 (manuscript). Figure S2: HPLC chromatograms of *S. nigra* flower extracts obtained by the shaking method. Peaks are numbered according to the compounds listed in Table 2 (manuscript). Solvents used: (A) EtOH-H_2_O mixture (1:1, *v*/*v*), (B) water, and (C) ethanol. Figure S3: HPLC chromatograms of *S. nigra* flower extracts obtained by the ultrasound-assisted method. Peaks are numbered according to the compounds listed in Table 2 (manuscript). Solvents used: (A) EtOH-H_2_O mixture (1:1, *v*/*v*), (B) water, and (C) ethanol. Figure S4: HPLC chromatograms of *S. nigra* flower extracts obtained by the accelerated solvent extraction method. Peaks are numbered according to the compounds listed in Table 2 (manuscript). Solvents used: (A) EtOH-H_2_O mixture (1:1, *v*/*v*), (B) water, and (C) ethanol. Figure S5: HPLC chromatograms of *S. nigra* flowers (Ukrainian origin) extracts obtained with EtOH-H_2_O mixture (1:1, *v*/*v*). Peaks are numbered according to the compounds listed in Table 2 (manuscript). Extraction methods used: (A) ASE (5 min, 100 °C, EtOH-H_2_O mixture (1:1, *v*/*v*)), (B) UAE (20 min, EtOH-H_2_O (1:1, *v*/*v*)), and (C) shaking (10 min, EtOH-H_2_O (1:1, *v*/*v*)). Figure S6: The influence of extraction technique and extracting solvent on the content of the assigned components of elderberry extracts. Figure S7: Calibration curves of rutin and chlorogenic acid standard samples. Table S2: Quantitative analysis of rutin and chlorogenic acid in *S. nigra* flower extracts obtained using different extraction methods determined by HPLC–PDA using external standard calibration.
