# Supplementary material for: From Elderflower to Bioactive Extracts: Phytochemical Characterization and Anti-Inflammatory Activity
Source: Molecules. 2026 Feb 5;31(3):561. doi: 10.3390/molecules31030561 (PMC12899531; doi:10.3390/molecules31030561)
Supplement: Supplementary file 1 [file molecules-31-00561-s001.zip › molecules-4039606-supplementary.pdf]

SUPPLEMENTARY FILE

Table S1. MS/MS chromatograms with fragmentation of identified compounds

**Quinic acid**
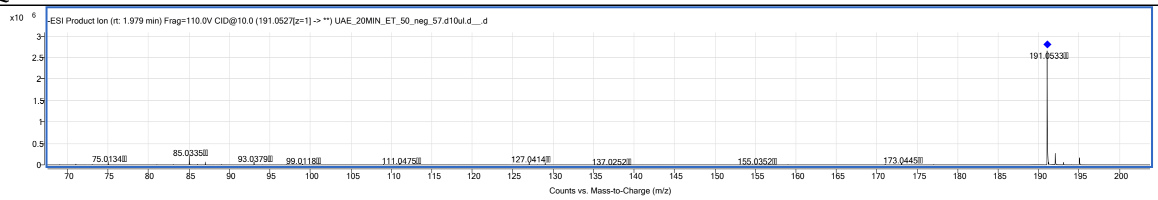
**Citric acid**
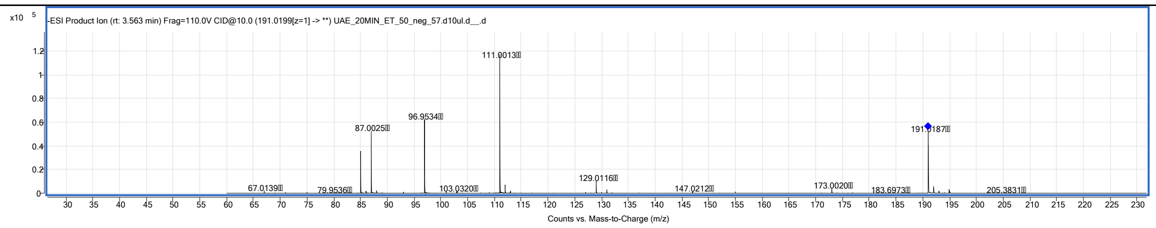
**Protocatechuic acid**
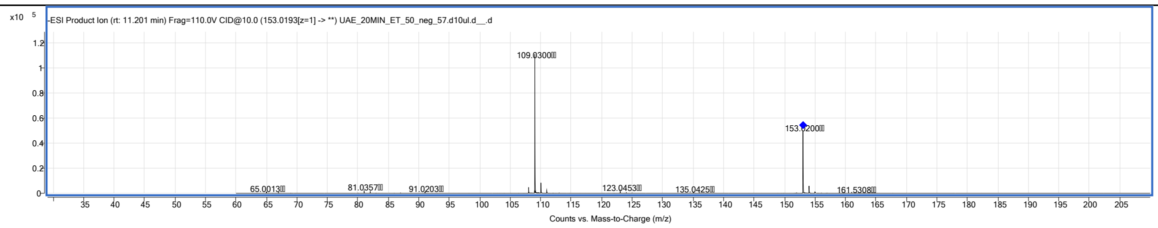
**Clorogenic acid isomer 1**
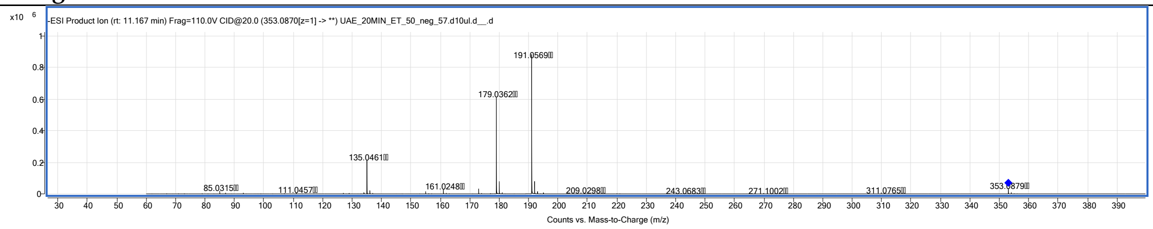
**Clorogenic acid isomer 2**
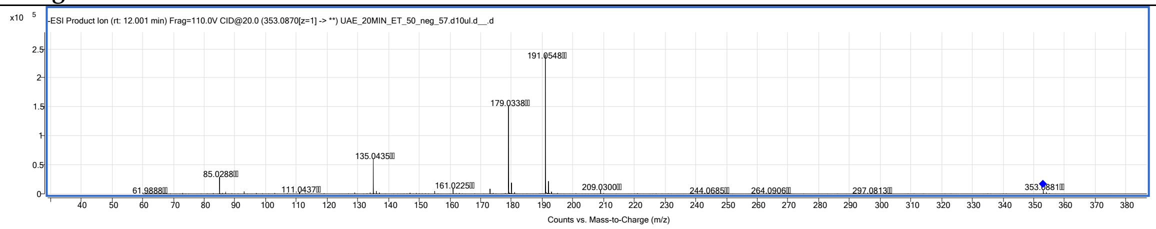
**3-Caffeoylquinic acid (Chlorogenic acid)**
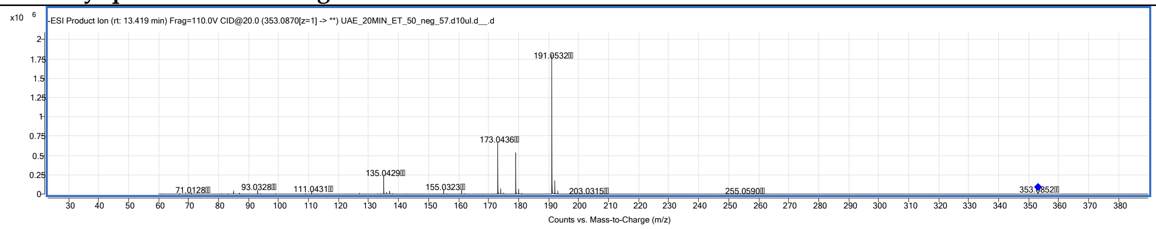
**Coumaroylquinic acid**

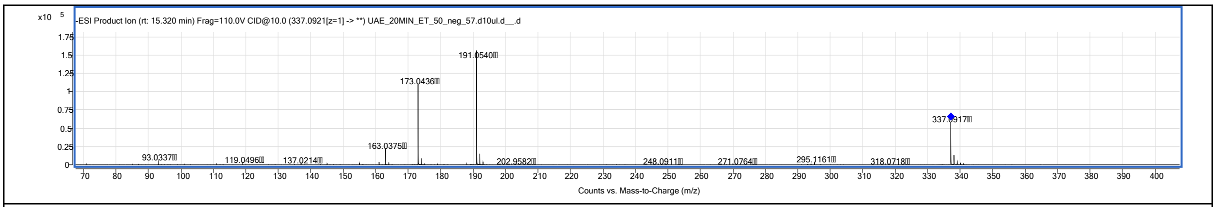

Rutin

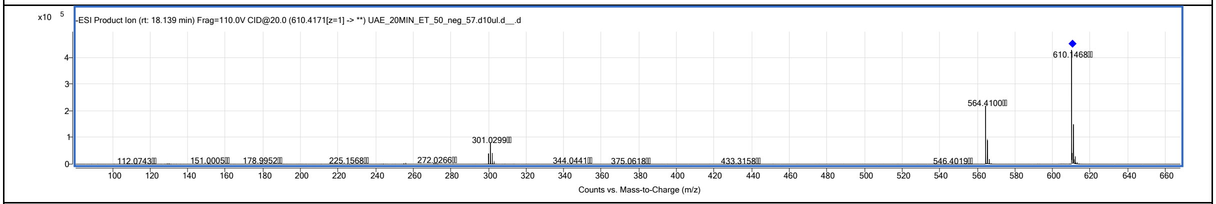

3',5'-Diprenylliquiritigenin

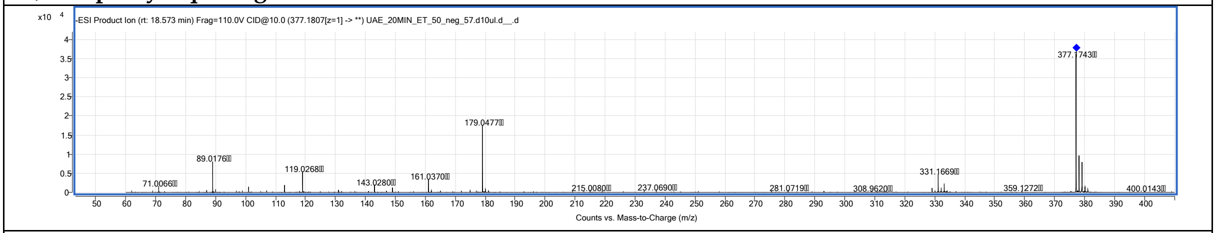

Quercetin 3-*D*-glucoside

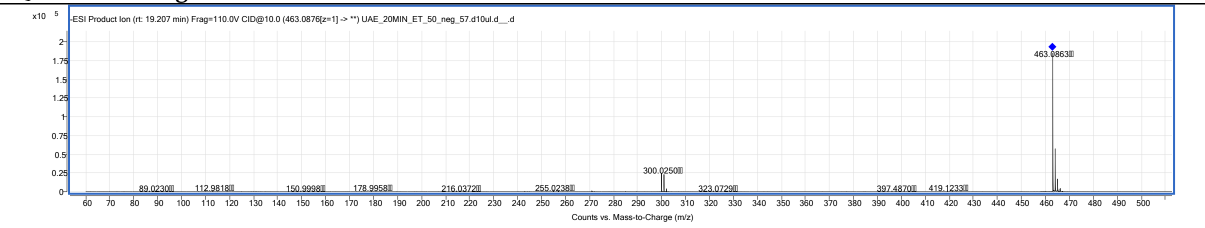

Kaempferol-3-*O*-rutinoside

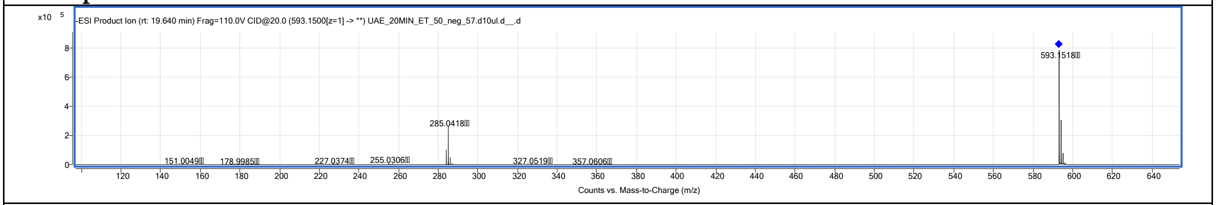

Isorhamnetin 3-*O*-rutinoside

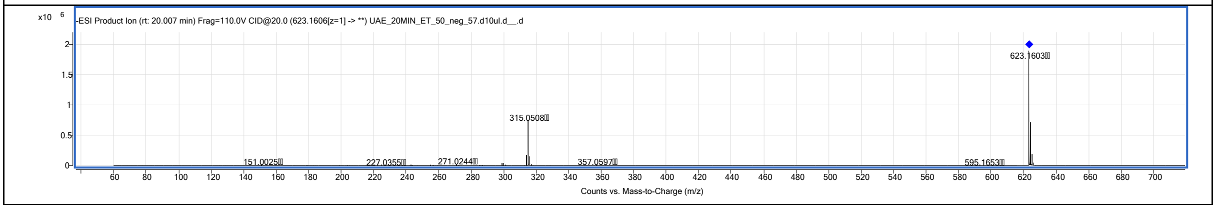

Quercetin 3-*O*-(6''-*o*-malonyl)-beta-*D*-glucoside

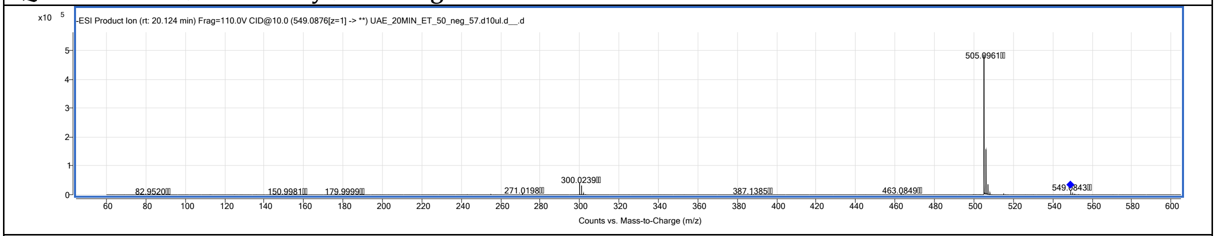

Kaempferol 3-*O*-glucoside

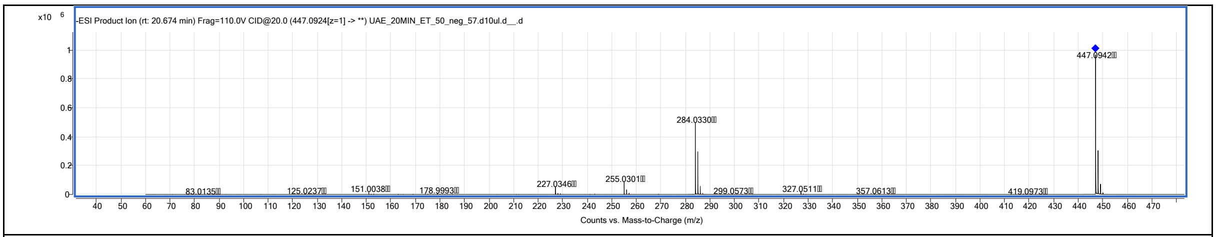

Dicaffeoylquinic acid isomer 1

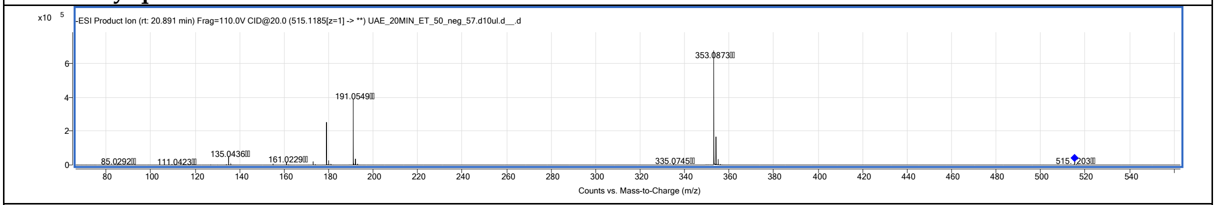

Isorhamnetin-3-*O*-glucoside

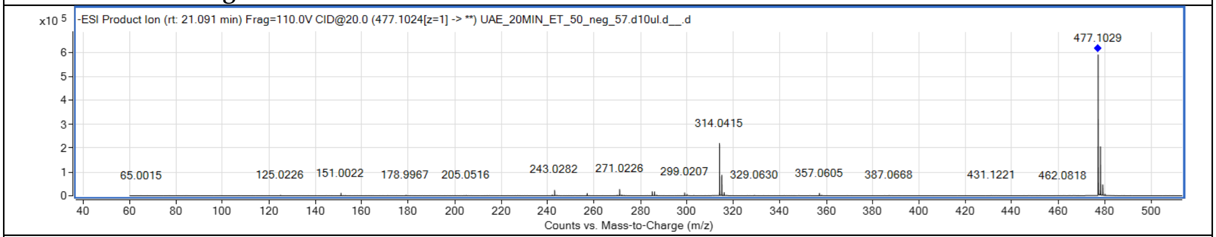

Dicaffeoylquinic acid isomer 2

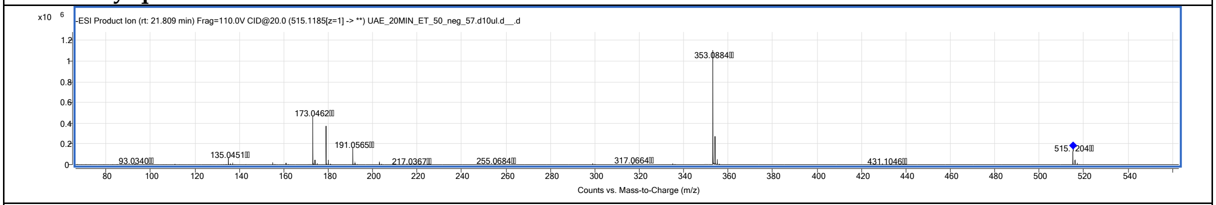

Spermidine isomer 1

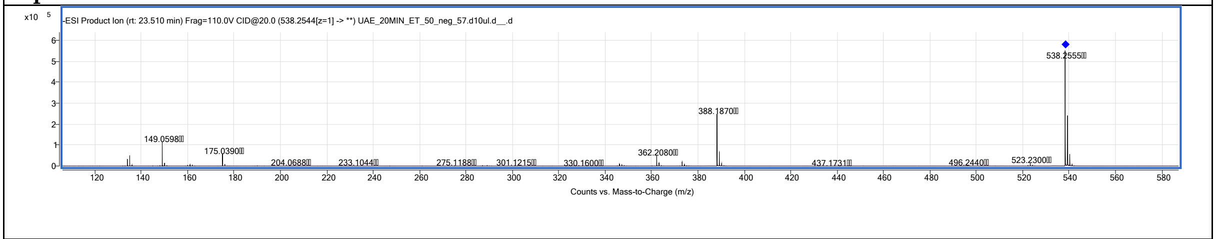

3-O-p-coumaroyl-4-O-caffeoylquinic acid isomer 1

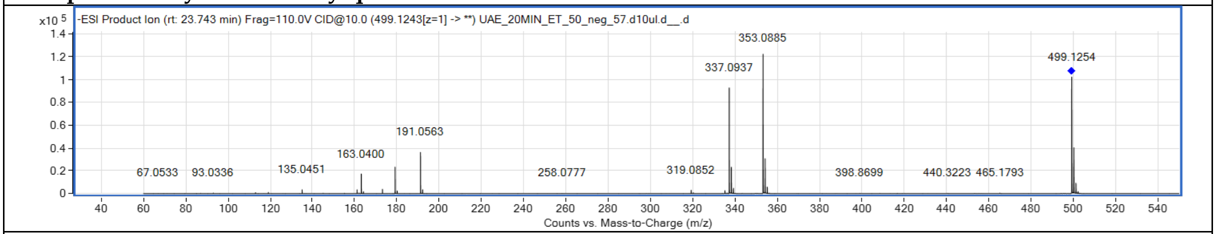

3-*O*-p-coumaroyl-4-*O*-caffeoylquinic acid isomer 2

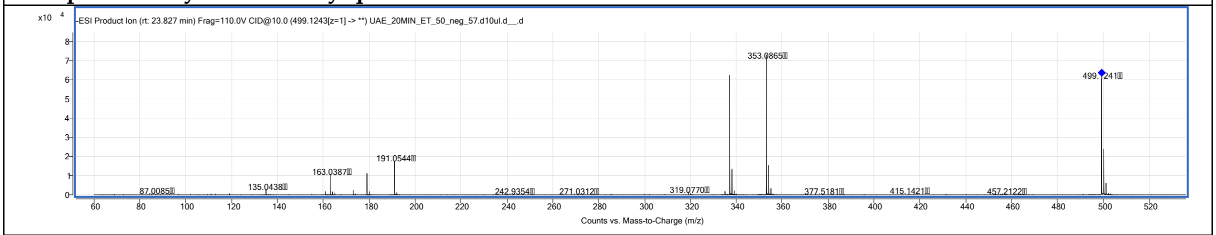

Spermididne isomer 2

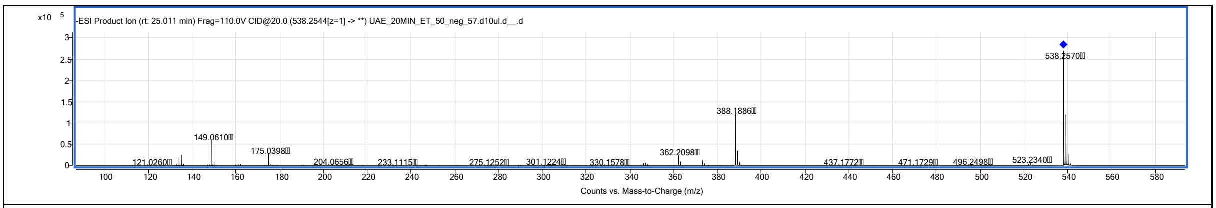

Malyngic acid

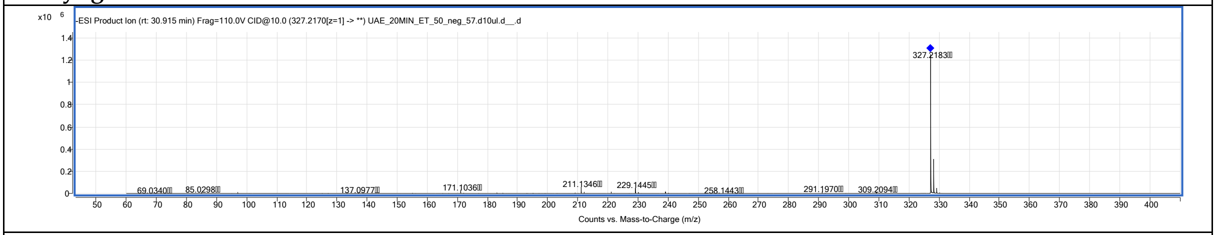

Naringenin

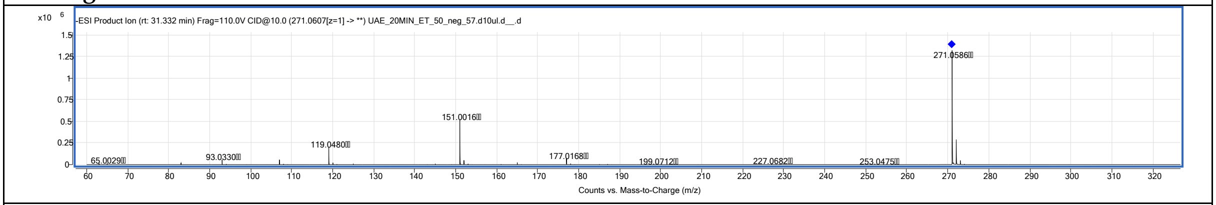

Pinellic acid

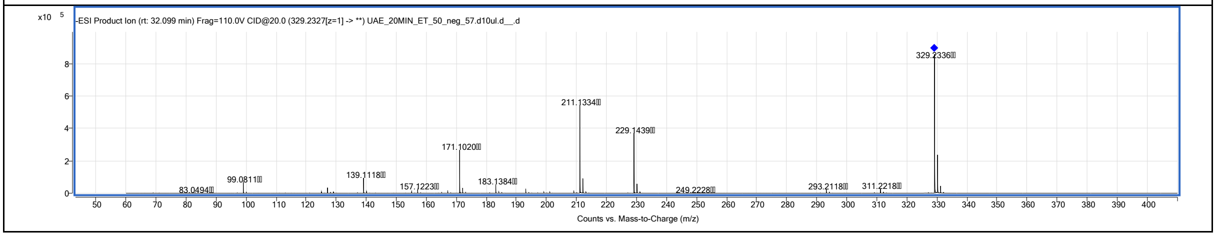

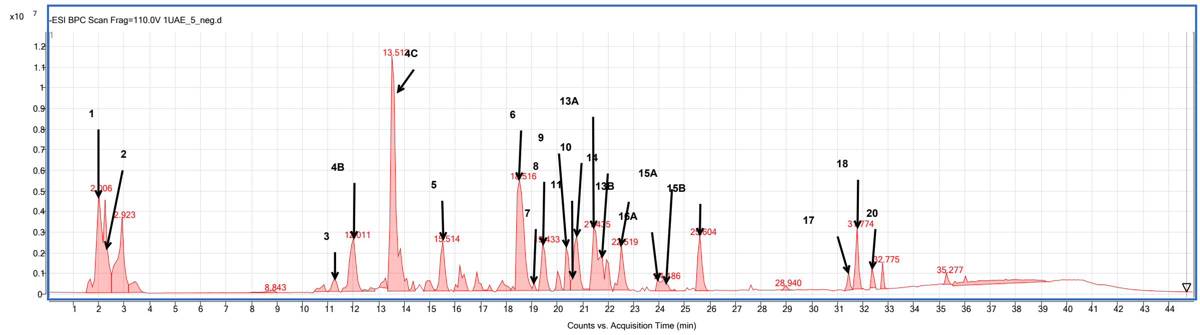

Figure S1. Base peak chromatogram (BPC) obtained in the negative ionization mode (HPLC-ESI-QTOF-MS/MS) from the elderberry flower (Ukrainian origin) extracted in ultrasounds for 20 min using EtOH-H_2O mixture (1:1, v/v). The peaks are numbered according to the compounds listed in Table 2 (manuscript).

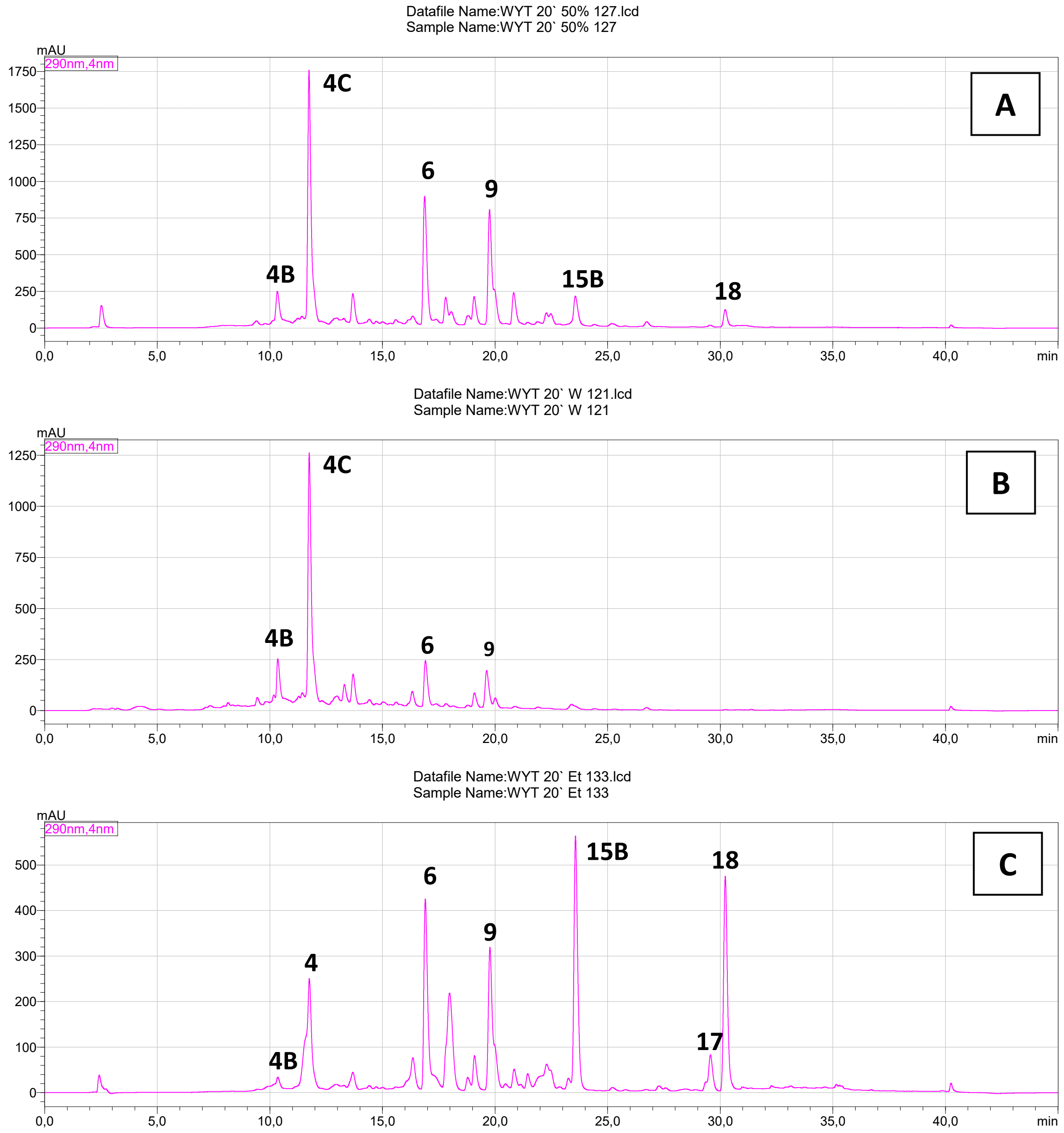

Figure S2. HPLC chromatograms of *S. nigra* flowers extracts obtained by the shaking method. Peaks are numbered according to the compounds listed in Table 2 (manuscript). Solvents used: (A) EtOH-H_2O mixture (1:1, v/v), (B) water, and (C) ethanol

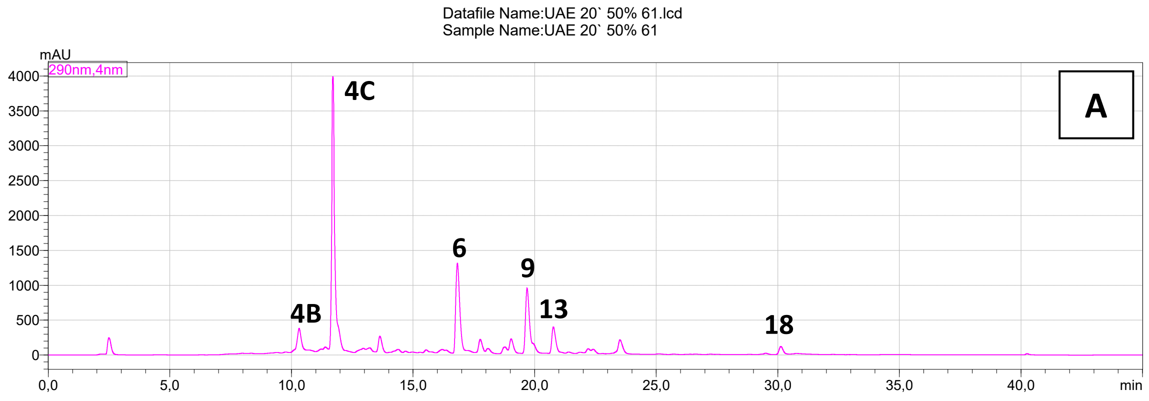

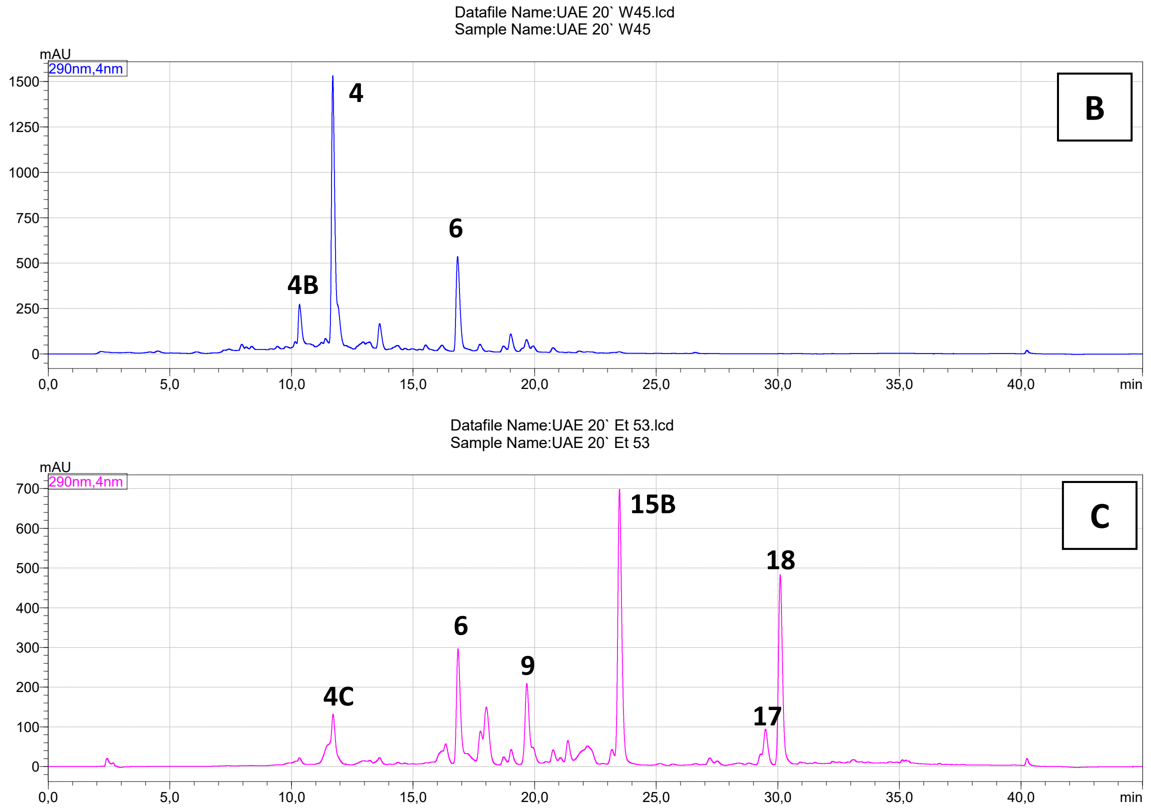

Figure S3. HPLC chromatograms of *S. nigra* flowers extracts obtained by the ultrasound assisted method. Peaks are numbered according to the compounds listed in Table 2 (manuscript). Solvents used: (A) EtOH-H_2O mixture (1:1, v/v), (B) water, and (C) ethanol

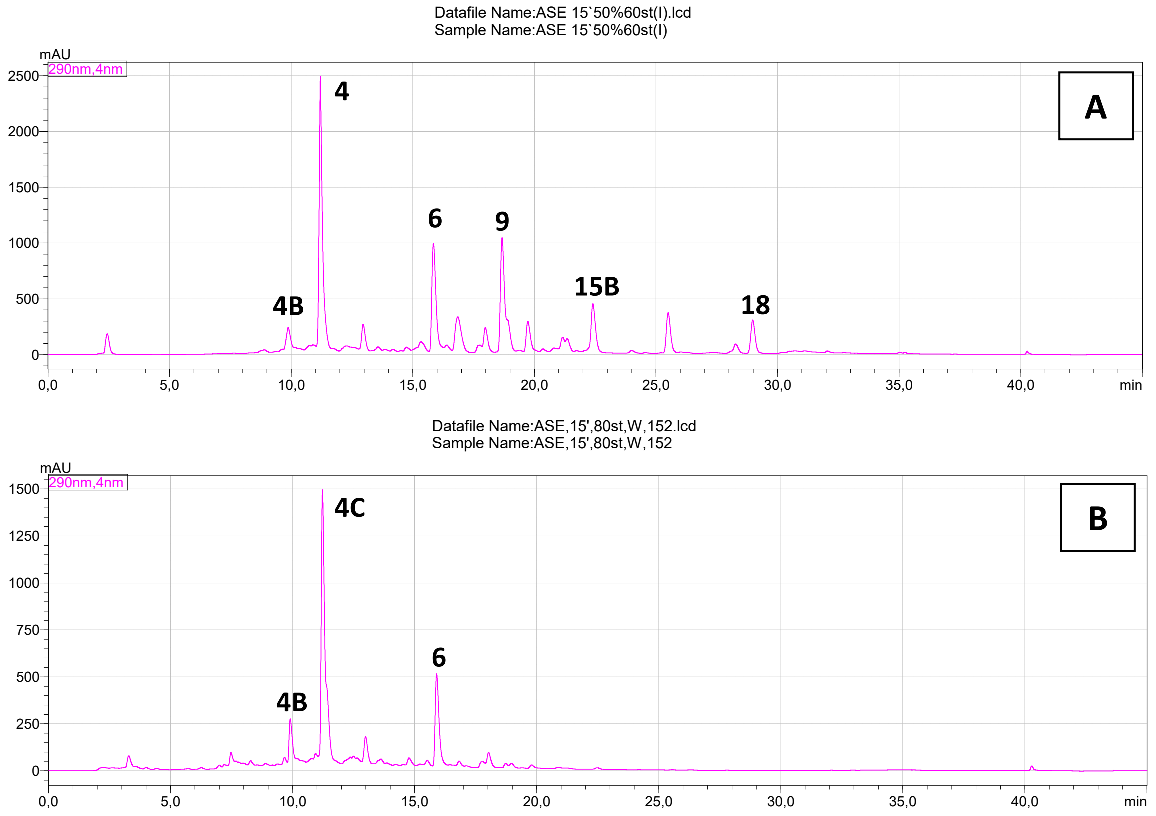

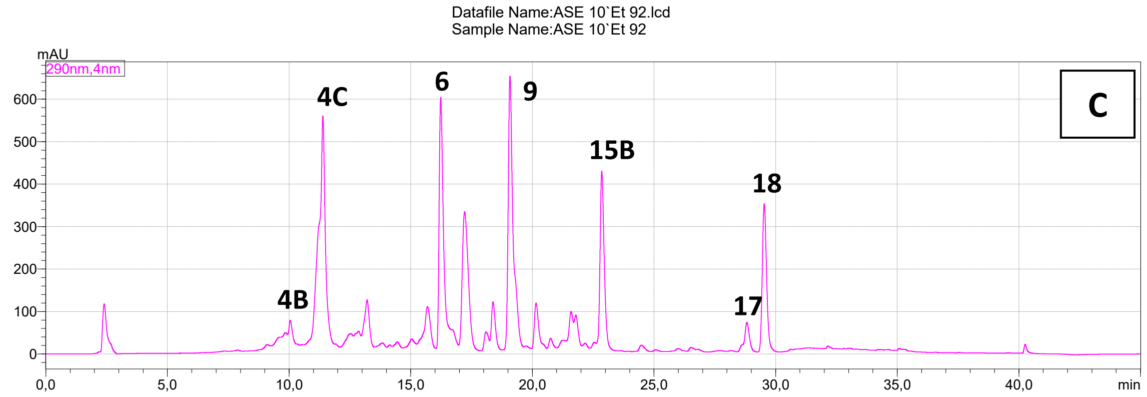

Figure S4. HPLC chromatograms of *S. nigra* flowers extracts obtained by the accelerated solvent extraction method. Peaks are numbered according to the compounds listed in Table 2 (manuscript). Solvents used: (A) EtOH-H_2O mixture (1:1, v/v), (B) water, and (C) ethanol

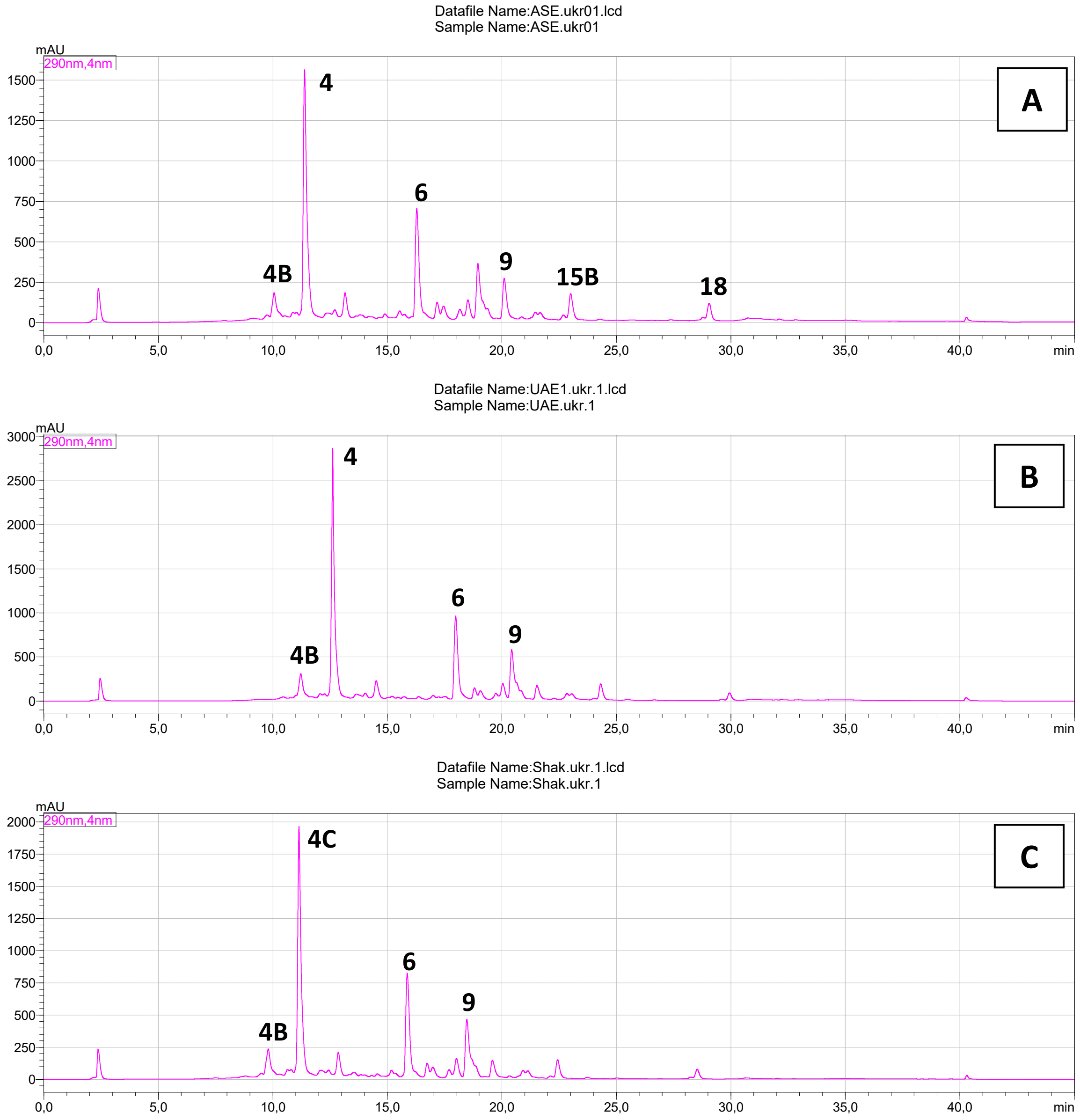

Figure S5. HPLC chromatograms of *S. nigra* flowers (Ukrainian origin) extracts obtained with EtOH-H_2O mixture (1:1, v/v). Peaks are numbered according to the compounds listed in Table 2 (manuscript). Extraction methods used:

(A) ASE (5min, 100°C, EtOH-H_2O mixture (1:1, v/v)), (B) UAE (20min, EtOH-H_2O (1:1, v/v)), and (C) Shaking (10min, EtOH-H_2O (1:1, v/v))

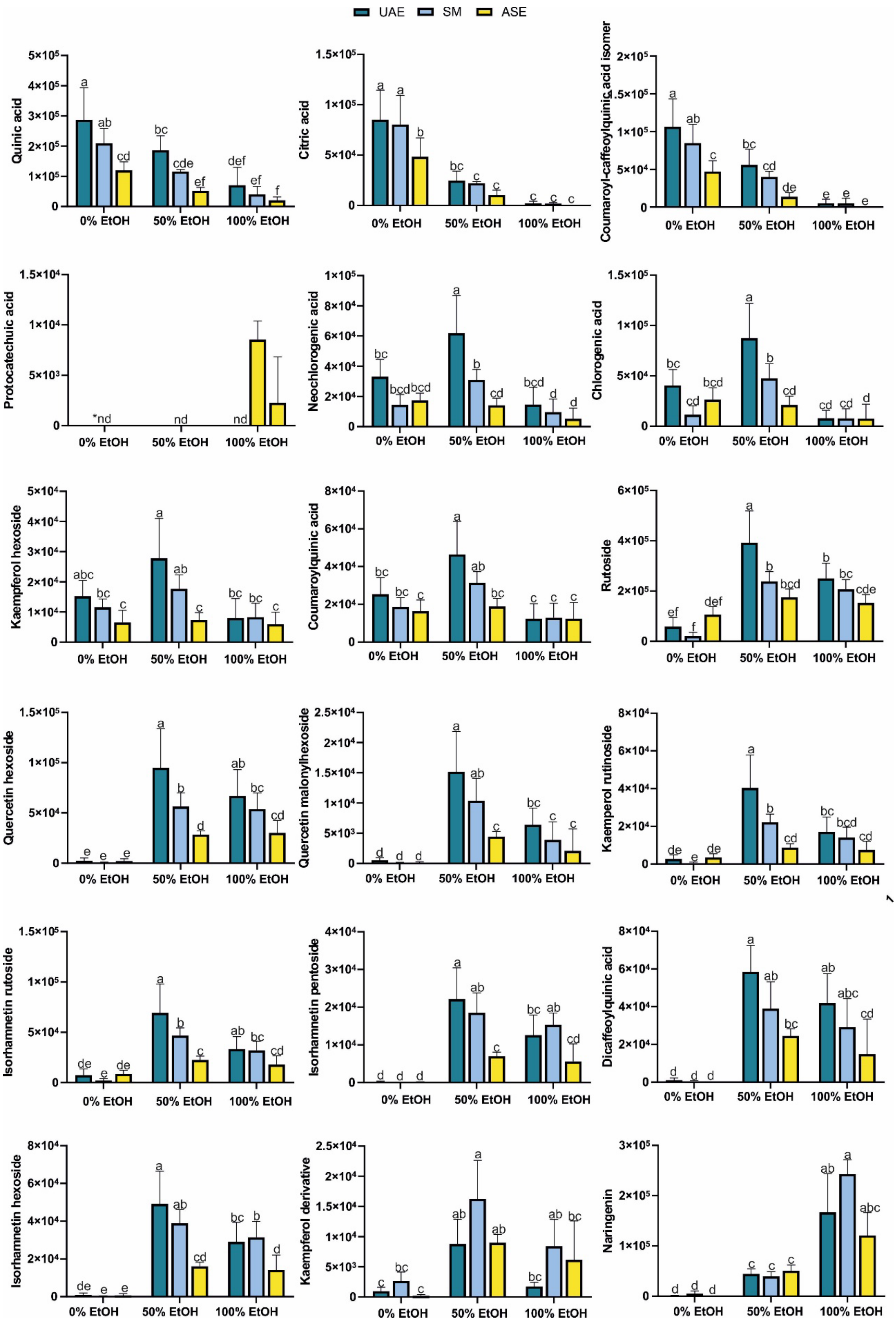

Figure S6. The influence of extraction technique and extracting solvent on the content of the assigned components of elderberry extracts.

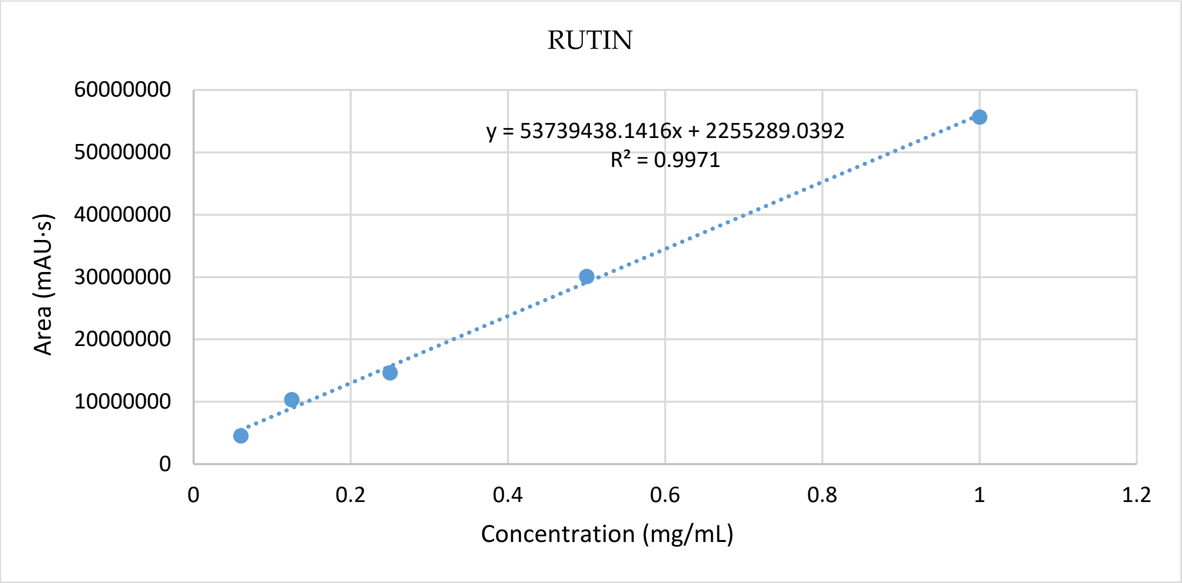

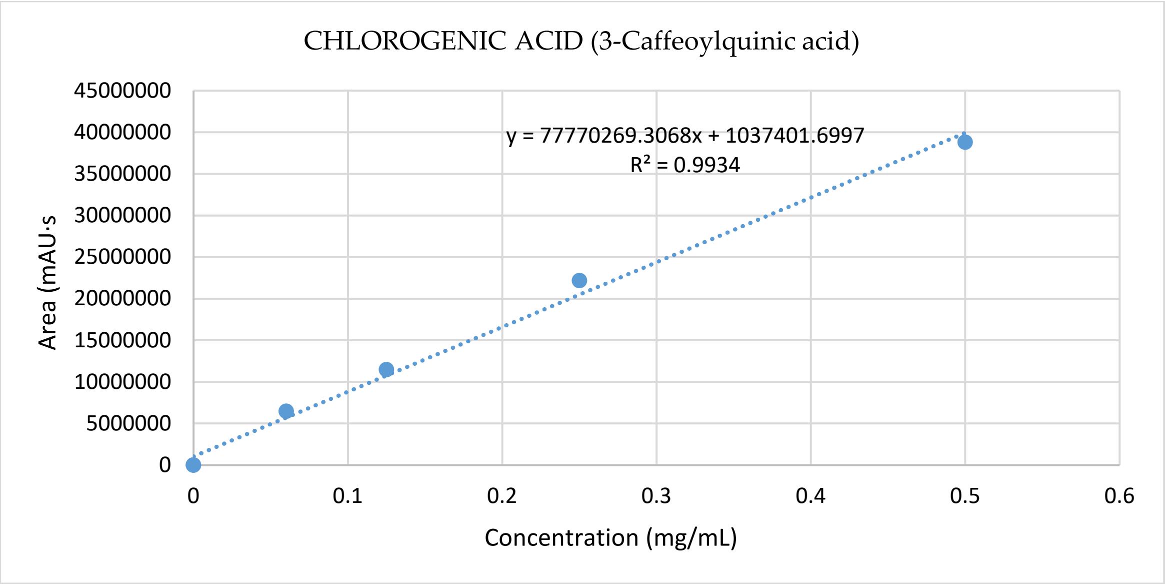

Figure S7 Calibration curves of rutin and chlorogenic acid standard samples

Table S2. Quantitative analysis of rutin and chlorogenic acid in *S. nigra* flower extracts obtained using different extraction methods determined by HPLC–PDA using external standard calibration.

Extract ID	Internal ID	Extract method	Rutin					Chlorogenic acid				
			mg/ml	%	Mean (%)	SD (%)	RSD (%)	mg/ml	%	Mean (%)	SD (%)	RSD (%)
	70	UAE, 30 min, EtOH	0.1237	2.47	1.76	0.62	0.35	0.1427	2.85	1.26	1.41	1.11
	73		0.0730	1.46				0.0366	0.73			
11	74		0.0671	1.34				0.0099	0.20			
8	50	UAE, 20min, EtOH	0.0585	1.17	0.80	0.34	0.43	0.0260	0.52	0.13	0.34	2.49
	53		0.0249	0.50				-0.0040	-0.08			
	54		0.0367	0.73				-0.0018	-0.04			
5	29	UAE 10 min, EtOH	0.0176	0.35	0.61	0.91	1.50	0.0188	0.38	0.64	0.89	1.38
	31		-0.0075	-0.15				-0.0038	-0.08			
	26		0.0812	1.62				0.0817	1.63			
	7	UAE, 5 min, EtOH	0.0622	1.24	-0.08	1.14	n/a	0.0582	1.16	0.46	0.61	1.33
	11		-0.0369	-0.74				0.0051	0.10			
2	9		-0.0369	-0.74				0.0056	0.11			
	77	UAE, 30min, EtOH-H2O	0.1582	3.16	2.32	1.31	0.57	0.3097	6.19	4.52	2.68	0.59
	80		0.1498	3.00				0.2963	5.93			
12	83		0.0405	0.81				0.0715	1.43			
9	57	UAE, 20 min, EtOH-H2O	0.1309	2.62	4.87	2.45	0.50	0.2043	4.09	8.22	4.02	0.49
	60		0.3743	7.49				0.6058	12.12			
	61		0.2256	4.51				0.4235	8.47			
6	34	UAE, 10min, EtOH-H2O	0.1291	2.58	2.52	0.45	0.18	0.1967	3.93	4.13	0.43	0.11
	37		0.1463	2.93				0.2314	4.63			
	42		0.1021	2.04				0.1915	3.83			
	6	UAE, 5min, EtOH-H2O	0.1466	2.93	3.46	0.46	0.13	0.2064	4.13	4.77	0.57	0.12
	3		0.1825	3.65				0.2605	5.21			

1	1		0.1894	3.79				0.2493	4.99			
10	64	UAE, 30min, H2O	0.0622	1.24	1.17	0.18	0.15	0.2351	4.70	3.98	0.66	0.16
	65		0.0484	0.97				0.1706	3.41			
	68		0.0648	1.30				0.1919	3.84			
7	44	UAE, 20min, H2O	0.0252	0.50	0.93	0.37	0.40	0.1715	3.43	3.38	0.04	0.01
	45		0.0551	1.10				0.1684	3.37			
	48		0.0587	1.17				0.1677	3.35			
	21	UAE, 10min, H2O	0.0420	0.84	1.17	0.42	0.36	0.1161	2.32	3.47	0.99	0.29
	20		0.0516	1.03				0.2005	4.01			
4	23		0.0821	1.64				0.2031	4.06			
3	13	UAE, 5min, H2O	0.1237	1.34	2.96	0.07	0.02	0.2063	4.13	0.24	0.03	0.12
	2		0.1827	3.65				0.2416	4.83			
	17		0.1939	3.88				0.2629	5.26			
	150	Shaking maceration, 30 min; EtOH	0.0269	0.54	0.88	0.30	0.34	0.0413	0.83	0.35	0.41	1.16
	152		0.0528	1.06				0.0050	0.10			
24	154		0.0530	1.06				0.0068	0.14			
	134	Shaking maceration, 20 min; EtOH	0.0642	1.28	0.78	0.52	0.67	0.0225	0.45	0.11	0.57	4.96
21	137		0.0121	0.24				-0.0271	-0.54			
	133		0.0408	0.82				0.0217	0.43			
	115	Shaking maceration; 10min; EtOH	0.0421	0.84	0.69	0.14	0.21	0.0269	0.54	0.22	0.31	1.42
	116		0.0279	0.56				0.0103	0.21			
18	118		0.0332	0.66				-0.0042	-0.08			
15	97	Shaking maceration; 5 min; EtOH	0.0311	0.62	0.73	0.15	0.21	0.0040	0.08	0.05	0.07	1.56
	99		0.0450	0.90				-0.0019	-0.04			
	101		0.0331	0.66				0.0051	0.10			
	146	Shaking maceration; 30 min; EtOH-H2O	0.1052	2.10	2.55	0.58	0.23	0.1892	3.78	4.56	0.99	0.22
	145		0.1601	3.20				0.2836	5.67			
23	148		0.1171	2.34				0.2107	4.21			

20	127	Shaking maceration; 20min; EtOH-H2O	0.1386	2.77	2.91	0.14	0.05	0.2049	4.10	4.71	0.53	0.11
	129		0.1526	3.05				0.2540	5.08			
	130		0.1447	2.89				0.2477	4.95			
	109	Shaking maceration; 10 min; EtOH-H2O	0.1462	2.92	3.20	0.37	0.11	0.2065	4.13	4.71	0.50	0.11
17	110		0.1537	3.07				0.2493	4.99			
	113		0.1809	3.62				0.2509	5.02			
14	91	Shaking maceration; 5min; EtOH-H2O	0.1384	2.77	2.76	0.03	0.01	0.2028	4.06	3.99	0.29	0.07
	90		0.1362	2.72				0.1836	3.67			
	95		0.1391	2.78				0.2123	4.25			
22	138	Shaking maceration; 30 min; H2O	0.0170	0.34	0.00	0.30	n/a	0.0695	1.39	1.08	0.49	0.45
	141		-0.0110	-0.22				0.0663	1.33			
	143		-0.0063	-0.13				0.0259	0.52			
19	121	Shaking maceration; 20 min; H2O	0.0028	0.06	0.16	0.11	0.67	0.1369	2.74	2.37	0.40	0.17
	122		0.0137	0.27				0.1218	2.44			
	125		0.0077	0.15				0.0969	1.94			
16	103	Shaking maceration; 10 min; H2O	0.0153	0.31	0.55	0.23	0.41	0.0744	1.49	2.22	0.66	0.30
	105		0.0296	0.59				0.1376	2.75			
	107		0.0378	0.76				0.1218	2.44			
	84	Shaking maceration; 5 min; H2O	-0.0038	-0.08	-0.07	0.14	n/a	0.0730	1.46	1.40	0.14	0.10
	86		-0.0099	-0.20				0.0621	1.24			
13	88		0.0038	0.08				0.0752	1.50			
	105	ASE 15 min, 100°C; EtOH	0.2007	4.01	3.25	1.60	0.49	0.1693	3.39	2.82	0.84	0.30
42	108		0.2168	4.34				0.1610	3.22			
39	93	ASE 15 min, 80°C; EtOH	0.0707	1.41	1.55	0.27	0.18	0.0930	1.86	1.71	0.13	0.08
	95		0.0688	1.38				0.0833	1.67			
36	80	ASE 15 min, 60°C; EtOH	0.0935	1.87	2.39	0.65	0.27	0.0802	1.60	2.11	1.09	0.52
	83		0.1088	2.18				0.0683	1.37			
41	102		0.1563	3.13	2.54	0.68	0.27	0.1679	3.36	2.61	0.81	0.31

	104	ASE 10 min, 100°C; EtOH	0.1348	2.70				0.1366	2.73			
38	90	ASE 10 min, 80°C; EtOH	0.0898	1.80	1.97	0.15	0.08	0.0872	1.74	1.75	0.29	0.17
	92		0.1018	2.04				0.1026	2.05			
	75	ASE 10 min, 60°C; EtOH	0.1034	2.07	1.92	0.21	0.11	0.0734	1.47	1.57	0.50	0.32
35	77		0.0841	1.68				0.0560	1.12			
	98	ASE 5 min, 100°C; EtOH	0.1001	2.00	1.98	0.26	0.13	0.1054	2.11	2.08	0.26	0.12
40	100		0.1117	2.23				0.1163	2.33			
	85	ASE 5 min, 80°C; EtOH	0.0854	1.71	1.81	0.13	0.07	0.0906	1.81	1.76	0.30	0.17
37	88		0.0977	1.95				0.1010	2.02			
34	66	ASE 5 min, 60°C; EtOH	0.0890	1.78	1.85	0.14	0.08	0.0716	1.43	2.10	1.18	0.56
	72		0.1007	2.01				0.0703	1.41			
33	64	ASE 15 min, 100 °C; EtOH-H2O	0.0881	1.76	1.00	1.53	1.53	0.1730	3.46	4.64	1.67	0.36
	63		-0.0381	-0.76				0.3274	6.55			
	58	ASE 15 min, 80 °C; EtOH-H2O	0.0994	1.99	2.03	0.16	0.08	0.1956	3.91	3.74	0.35	0.09
32	59		0.0944	1.89				0.1665	3.33			
	55	ASE 15 min, 60 °C; EtOH-H2O	0.1104	2.21	1.93	0.72	0.37	0.1985	3.97	2.90	3.18	1.10
31	54		0.0560	1.12				0.2697	5.39			
	47	ASE; 10min, 100°C EtOH-H2O	0.1236	2.47	2.64	0.43	0.16	-0.0339	-0.68	-0.68	0.00	0.00
30	51		0.1166	2.33				-0.0338	-0.68			
29	40	ASE; 10min, 80°C EtOH-H2O	0.1565	3.13	2.05	1.98	0.96	-0.0338	-0.68	-0.67	0.01	n/a
	43		0.1630	3.26				-0.0334	-0.67			
28	34	ASE; 10min, 60°C EtOH-H2O	-0.0114	-0.23	0.81	1.58	1.97	-0.0327	-0.65	1.11	3.08	2.77
	37		0.0008	0.02				-0.0341	-0.68			
25	28	ASE, 5min, 100°C, EtOH-H2O	0.1315	2.63	2.73	0.08	0.03	0.2335	4.67	5.01	0.53	0.11
	20		0.1395	2.79				0.2810	5.62			
27	14		0.1378	2.76	2.53	0.35	0.14	0.2365	4.73	4.31	0.72	0.17

	18	ASE, 5min, 80°C, EtOH-H2O	0.1360	2.72				0.2364	4.73			
26	31	ASE, 5min,60°C, EtOH-H2O	0.1063	2.13	2.26	0.25	0.11	0.1738	3.48	4.14	0.61	0.15
	25		0.1275	2.55				0.2121	4.24			
45	120	ASE, 15min, 100°C, H2O	0.1053	2.11	1.79	0.34	0.19	0.2344	4.69	4.31	0.73	0.17
	122		0.0921	1.84				0.2381	4.76			
	156	ASE, 15min, 80°C, H2O	0.0716	1.43	1.24	0.16	0.13	0.1735	3.47	3.70	0.37	0.10
51	152		0.0569	1.14				0.1751	3.50			
48	138	ASE, 15min, 60°C, H2O	0.0579	1.16	0.83	0.36	0.43	0.2060	4.12	3.44	0.71	0.21
	141		0.0225	0.45				0.1352	2.70			
	118	ASE, 10min, 100°C, H2O	0.0449	0.90	1.42	0.45	0.32	0.1752	3.50	4.08	0.52	0.13
44	115		0.0839	1.68				0.2256	4.51			
	150	ASE, 10min, 80°C, H2O	0.0843	1.69	1.41	0.26	0.18	0.2119	4.24	3.94	0.83	0.21
50	146		0.0587	1.17				0.1504	3.01			
47	134	ASE, 10min, 60°C, H2O	0.0680	1.36	1.71	0.63	0.37	0.2291	4.58	4.97	0.78	0.16
	133		0.0662	1.32				0.2936	5.87			
	113	ASE, 5min, 100°C, H2O	0.1221	2.44	2.04	0.79	0.39	0.2228	4.46	4.15	0.99	0.24
43	112		0.1271	2.54				0.2477	4.95			
	142	ASE, 5min, 80°C, H2O	0.0566	1.13	1.23	0.11	0.09	0.1527	3.05	3.67	0.54	0.15
49	129		0.0598	1.20				0.1989	3.98			
46	125	ASE, 5min, 60°C, H2O	0.0676	1.35	1.30	0.00	0.00	0.1996	3.99	0.19	0.01	0.05
	127		0.0628	1.26				0.1847	3.69			
Ukr UAE	UKR	ASE, 5min, 100°C, EtOH-H2O	0.1039	2.08	2,05	0,03	0,01	0.1899	3.80	3,74	0,06	0,02
			0.1018	2.04				0.1856	3.71			
			0.1011	2.02				0.1862	3.72			
Ukr UAE	UKR	UAE, 20min, EtOH-H2O	0.1681	3.36	2,93	0,65	0,22	0.2971	5.94	5,00	1,25	0,25
			0.1222	2.44				0.2089	4.18			
			0.1486	2.97				0.2445	4.89			
Ukr Shak	UKR	Shaking maceration, 10min, EtOH-H2O	0.1327	2.65	2,70	0,11	0,04	0.2292	4.58	4,80	0,31	0,07
			0.1249	2.50				0.2514	5.03			

			0.1481	2.96				0.2389	4.78			

Note: The concentrations (mg/mL) were calculated from the peak areas using the linear regression equation obtained from the calibration curve of the corresponding standard. Mean (%) represents the average percentage of rutin/ chlorogenic acid in the extract obtained by the respective extraction method, calculated from three (UAE, shaking maceration) or two (ASE) independent replicates. SD – standard deviation; RSD (%) – relative standard deviation; n/a – not applicable; The number of replicates was n = 3 for UAE and shaking, and n = 2 for ASE. UAE – Ultrasonic-Assisted Extraction; ASE – accelerated extraction; EtOH – pure ethanol; H2O – water; EtOH-H2O – ethanol-water mixture (1:1, v/v); Extract ID refers to the extract numbering system introduced in the manuscript, whereas Internal ID corresponds to the original internal laboratory coding used during sample preparation and analysis. Ukr UAE, Ukr Shak, Ukr ASE - extracts prepared from Ukrainian raw material by the aforementioned methods.
